# Re-validation of the Cancer Research UK Cancer Awareness Measure ‘plus’ (CAM+): a study protocol

**DOI:** 10.3389/fpubh.2026.1841597

**Published:** 2026-06-24

**Authors:** Maja Nikšić, Kate Hamilton-West, Lindsay J. L. Forbes, Kirstie Osborne, Leia Brasnell, Sabina Hulbert, Kate Day, Victoria Whitelock, Sarah Hotham

**Affiliations:** 1 Centre for Health Services Studies, University of Kent, Canterbury, United Kingdom; 2 Health Policy and Evidence Department, Cancer Research UK, London, United Kingdom

**Keywords:** cancer awareness, early diagnosis, early prevention behaviour, questionnaire, validation

## Abstract

**Background:**

The Cancer Research UK Cancer Awareness Measure (CRUK CAM) was introduced in 2008 as the first psychometrically validated survey of public attitudes, awareness and behaviours across cancer prevention, screening and early diagnosis. CRUK regularly collects CAM data in the UK to inform strategic planning. The measure has been modified over time in response to changes in the research, policy and practice landscape. Our aim is to revalidate the most recent CAM ‘Plus’ (CAM+) to ensure the measure remains accurate, reliable, valid, and relevant over time.

**Methods:**

There will be four phases, running from 2023 to 2026, with the CAM + being revised after each phase. Phase 1 will start by auditing the CAM + against a behaviour change framework, i.e., the COM-B, postulating that to engage in a Behaviour (B), a person must have the Capability (C) and Opportunity (O) to exhibit that behaviour, as well as the Motivation (M) to demonstrate it at a specific moment. This phase will also include (a) scoping literature reviews; (b) a face validity assessment, to check CRUK and scientific evidence needs and identify gaps in the survey; and, (c) a readability assessment using the Hemingway App to check for long complex sentences. Phase 2 will focus on content validity, using two stages of cognitive interviews with members of the public to assess comprehension and clarity. The first stage will use a ‘Think Aloud’ methodology to identify potential problem areas; while the second stage will use the four-stage model of cognitive interviewing to understand how people comprehend, retrieve, judge, and respond to questions. In Phase 3, we will conduct exploratory and confirmatory factor analysis to inform further refinements to the measure. In Phase 4, we will assess: (a) construct validity, by comparing responses of cancer experts and non-cancer experts; (b) reliability, using the test–retest approach; and (c) responsiveness, using sensitivity to change to questions in CAM+, following a brief intervention. Throughout the revalidation process we will be adhering to the standards set out in the COSMIN guidelines.

## Introduction

### Description of Cancer Awareness Measure (CAM)

The Cancer Awareness Measure (CAM) has been established as a powerful public health tool for shaping cancer policy and interventions to improve earlier symptomatic presentation and screening uptake (1); and, a potential to impact cancer survival (2, 3). It was the first psychometrically validated measure of cancer awareness, attitudes and behaviours among the UK general public (4). It was developed by Cancer Research UK (CRUK), University College London, Kings College London and Oxford University in 2008. The measure covers a range of topics, from recognition of cancer symptoms and risk factors, barriers to seeking medical attention to knowledge of screening programmes in the UK. From 2008 onwards, CRUK has regularly collected CAM/CAM + data (5).

### CAM modifications

The CAM has been modified several times by CRUK over the years, in response to emerging evidence and evidence gaps, to include more topics and expand the list of potential cancer signs/symptoms (5). Since 2014 the measure has been referred to as CAM ‘Plus’ (CAM+), to recognise changes to the original survey instrument (5).

In 2017, CAM + data were collected through both face-to-face and online methods to compare outcomes and assess differences based on the mode of data collection. As there were no significant differences in survey findings between the two methods (6), the CAM + measure shifted to an online-only format from 2019 onwards.

Substantial modifications of the CAM + measure happened during the COVID-19 pandemic. More questions were added on help-seeking for cancer symptoms, the impact of COVID-19, and experiences during the latest GP appointment (7, 8). For example, four symptoms of cancer were added, such as: “coughing up blood,” “shortness of breath,” “tired all the time,” and “a change to an existing cough.” A separate category about confidence in noticing symptoms was introduced: “How confident are you that you would notice a symptom of lung cancer in yourself?” COVID-19 specific questions included: “I am confident that I would be safe from coronavirus if I needed to attend an appointment at a hospital (or my GP surgery)”; “I’m worried about delays to cancer screening caused by coronavirus” (8).

Furthermore, modifications to the 2019 CAM + included changes in format or wording of questions (9). One key motivation for rewording was to reduce the bias introduced by using the conditional tense, for example: “I *would be worried* about wasting the doctor’s time,” from 2019 onwards was changed to the indicative: “I *worried* about wasting the healthcare professional’s time” (9). This change reflected evidence that hypothetical questions about behaviour overestimate actual behaviour, as people express greater willingness to engage in preventive measures when presented with hypothetical situations than they do in real-life (10, 11). The choice of words changed from “doctor’s time” to “the healthcare professional’s time” (9), making it more inclusive by acknowledging the roles of nurses, physician associates, and other healthcare staff involved in patient care.

Some questions that were previously phrased in either present or future tense were rephrased in past tense. For example, “I find it difficult to get an appointment at a convenient time” (question used in CAM + from 2014 to 2017), from 2019 onwards became “I found it difficult to get an appointment at a convenient time” (5). Other modifications to the 2019 measure included changes to the answers’ format, i.e., moving from a 5-point Likert scale (Strongly agree to Strongly disagree) to binary answers only (tick box options: Yes/No) (9).

In the September 2023 version, a definition of ‘persistent’ was added’ to the questions about warning signs or symptoms of cancer: “persistent means does not go away” (12).

### Frequency of CAM data collection

From 2008 until 2019, data collection has continued at least every 2 years, and then every 6 months from 2020 to 2023, because of the need to understand the impact of the pandemic and support policy and action to mitigate its impact on cancer control (5).

### How CAM data have been used

The CAM results have been pivotal in informing cancer policy and practice, by identifying public knowledge gaps, understanding behaviour patterns and needs, and guiding the development of targeted interventions to promote early cancer detection and prevention (13). First, The need to improve cancer awareness and early presentation and diagnosis are acknowledged in the NHS Long Term Plan (14), and the Independent NHS England Cancer Taskforce (15).

Furthermore, CRUK use the data generated from CAM + to: (1) monitor, track and evaluate changes in public attitudes, awareness and behaviour; (2) identify priority topics and audiences; and, (3) inform CRUK’s public health engagement and behaviour change activities, e.g., health marketing campaigns (1).

Second, the CAM has been instrumental in shaping and evaluating interventions aimed at early cancer detection and prevention, especially the national *“Be Clear on Cancer”* campaigns (3, 16–18). Evaluations of these campaigns suggest that it is possible to raise awareness of early cancer symptoms, which may have an effect on increased help seeking, more urgent referrals by GPs, and higher likelihood of early-stage diagnosis (3, 16). Moreover, community-based awareness campaigns, such as the CRUK Cancer Awareness Roadshow, have used the CAM pre- and post-visit and have demonstrated impact on proactive health-seeking behaviours, and smoking cessation (19).

Third, the CAM was used as a foundation for site-specific CAMs, to enable assessment of the level of awareness, attitudes and behaviours for particular cancers (5). These have been validated for cancers of breast (20), bowel (21), lung (22), ovary and cervix (23), and blood (24). The site-specific CAMs have informed targeted interventions, such as the campaigns *Be Clear on Bowel Cancer* (25–27), *Be Clear on Lung Cancer* (3, 27, 28), and *Be Clear on Breast* Cancer (29, 30). However, these campaigns have been criticised for having exacerbated health inequalities: most failed to increase referrals among people from a lower socio-economic position (SEP), instead increasing referrals among the ‘worried well’ (25–27, 31).

Finally, CAM findings have been used by researchers to advance scientific understanding of cancer awareness, attitudes and behaviours. It has built a strong evidence base showing that some groups—the less affluent, older people and ethnic minorities in particular—have low cancer symptom awareness, poor knowledge of cancer risk factors, and increased barriers to seeking medical help; and report longer delays in seeking medical help (32–37). Questions remain about other underserved groups, for example, the LGBTQ+ population, people with visible and invisible disabilities and mental health disorders. Furthermore, the intersectionality perspective has not been explored—e.g., the combined effects of ethnicity, poor mental health, physical disability and poverty.

### Why is revalidation of the CAM important?

CRUK has carried out some activities to partially validate revised versions of the CAM, including cognitive testing with people affected by cancer (unpublished, personal communication, Victoria Whitelock, March 2025), but a comprehensive validation has not been carried out. Considering these efforts and the fact that the original CAM (2008) was validated, it is unlikely that the CAM + as a whole is measuring something completely different to the intended purpose.

Nevertheless, revalidation is important to check that items and completion instructions are clear, easy to understand and not subject to misinterpretation. Clarifying the underlying constructs measured in the current version of CAM + is also important, so that we can be sure that we are interpreting individual items accurately, for example: does it include measurement of constructs we think it does?; which items relate to each construct?; are any constructs missing?; are there redundant items or similar items that could be combined?

Re-validation can ensure that the CAM+ (1) continues to generate robust and reliable data to shape policy and practice to prevent cancer and promote early diagnosis. This protocol sets out the plans to revalidate the latest CAM + to ensure its relevance over time, accuracy, and effectiveness in measuring the UK general public’s cancer awareness, attitudes and behaviours; and evaluating interventions to promote it.

## Materials and methods

The latest CAM (CAM+) was developed by CRUK in September 2023 and collects data on the following topics:

- Help-seeking for potential cancer symptoms
- Experiences of seeking help
- Attitudes towards and experiences of remote consultations
- Barriers to help-seeking
- Prompts to help-seeking
- Re-presentation for persistent symptoms (including after tests)
- Facilitators of attending hospital tests
- COVID-19 safety concerns in several health settings
- Awareness of cancer risk factors
- Awareness of signs and signs/symptoms of cancer
- Past uptake of bowel, breast and cervical screening invitations
- Intentions to take part in bowel, breast and cervical screening
- Barriers to uptake of bowel, breast and cervical screening

Throughout the revalidation process, running from 2023 until 2026, we will adhere to the standards set out in the COSMIN guidelines (38). The revalidation process of the CAM + will incorporate four main phases.

### Phase 1: Literature review and face validity

Phase 1 will include a face validity assessment, scoping reviews of recent published literature and a COM-B audit (39, 40), which will check evidence needs, identify gaps in the measure, and inform further updates and refinements of CAM+. A readability assessment using the Hemingway Editor App will be used to identify and correct long complex sentences in CAM + that may not be understood by some audiences (41). The Hemingway Editor App will be used as a practical supplementary tool to identify potentially complex wording and long sentences. To support a more robust assessment of readability and accessibility, the Simple Measure of Gobbledygook (SMOG) readability index will also be used, as it is widely applied in health literacy and patient information research (42). As a result, we will modify identified sentences in several different ways. For example, where needed, we will (a) use plain language and improve their clarity, making them more concise and easier to understand; (b) minimize cognitive load, making the structure of sentences simpler and easier for participants to process and respond to accurately; and/or, (c) increase engagement, making the questions straightforward and easy to read to ensure people are willing to complete the measure (41, 43, 44). A revised CAM + will be developed based on the results of phase 1.

We will use the COM-B model as a theoretical framework for this project, because it helps researchers and practitioners understand the factors that influence behaviour change and identify potential targets for health interventions (39, 40).

The COM-B model includes three components:

1. Capability: This refers to the individual’s psychological and physical capacity to engage in a behaviour. It incorporates both physical abilities and psychological capabilities, such as knowledge, skills, and awareness.
2. Opportunity: This refers to the external factors that may facilitate or hinder behaviour, including environmental and social factors such as access to resources, social norms, and cultural influences.
3. Motivation: This refers to the individual’s mental processes that serve to initiate and direct behaviour. It incorporates both conscious and unconscious processes, such as beliefs, attitudes, emotions, etc.

The COM-B model proposes that interactions between these three components influence behaviour.

### Scoping review

A rapid scoping review will be undertaken to search for recent evidence on specific topics covered in the CAM+. The purpose of this work is to ensure the response options provided in the CAM + accurately reflect the current literature on enablers and barriers to screening, testing and help-seeking behaviour. The scoping review was restricted to literature published from 2020 onwards to capture changes in cancer-related help-seeking, screening participation, healthcare access, and symptom appraisal associated with the COVID-19 pandemic. Emerging evidence suggests that the pandemic substantially altered public engagement with healthcare services, including barriers to presentation, remote consultation experiences, and participation in cancer screening programmes, potentially influencing cancer awareness-related beliefs, attitudes, and behaviours (7, 45). The review therefore aimed to identify recent developments that may not have been reflected in earlier versions of CAM+. The review will focus solely on research using UK-based populations and use platforms EBSCOhost, PubMed and Web of Science. Search terms are outlined in Table 1.

**Table 1:** Scoping review search terms.

CAM sub-section topics	Search terms
Barriers and enablers to help seeking	(((Barrier* OR obstacle* OR challenge* OR obstruction* OR constraint* OR Enable* OR facilitate* OR support*) AND (“Help seeking” OR “help-seeking” OR “help seeking behaviour*” OR help) AND Cancer OR “Cancer sign*” OR “Cancer symptom*)))
Barriers to cervix screening	(((“Barriers to attending” OR “Barriers to participation”) AND (“cervix cancer screening” OR “cervical cancer screening”)))
Barriers to bowel screening	((“bowel cancer screening” OR “Bowel cancer detection” OR “FIT” OR Fecal immunochemical test”) AND (“barriers” OR “challenges” OR “Screening))
Barriers to breast screening	((“Breast cancer screening” OR “mammography”) AND (“barriers” OR “obstacles”) AND “UK”
Barriers and enablers to hospital tests	(((Barrier* OR obstacle* OR challenge* OR obstruction* OR constraint* OR Enable* OR facilitate* OR support*) AND (cancer OR “Cancer tests” OR) AND (hospital OR “secondary care” OR “hospital tests” OR Secondary Care OR pathway)))

Abstracts of papers will be reviewed to assess suitability. The findings will be cross-referenced with the response options already in the CAM + to check for replication and identify gaps, i.e., identifying an important barrier in the research that is not included in the CAM + .

### Face validity: using CRUK-based experts

We will conduct a Face Validity check, collecting data from CRUK experts in cancer research who use CAM/CAM + results, asking them whether the measure assesses what it is meant to assess. Experts will be asked to identify the questions that most frequently influence their work, or less used questions that could be removed; or to identify any gaps in coverage to inform the development of new questions in the CAM+. The feedback questionnaire will be designed by CRUK and hosted online. It will be circulated by the CRUK team across the relevant Directorates within the organisation.

### Phase 2: Cognitive interviews

In phase 2, cognitive interviews will assess item comprehension, interpretation and clarity of the revised CAM+. Overall, we will seek feedback on how to make questions more user-friendly, easier to understand or clearer, including both content and form of the questions.

This phase will seek to understand:

- Is the revised CAM + interpreted as intended?
- Are CAM + questions, response items and response options appropriate?
- Are there any gaps in coverage, e.g., any relevant barrier to help seeking that is missing?

**Figure 1:**
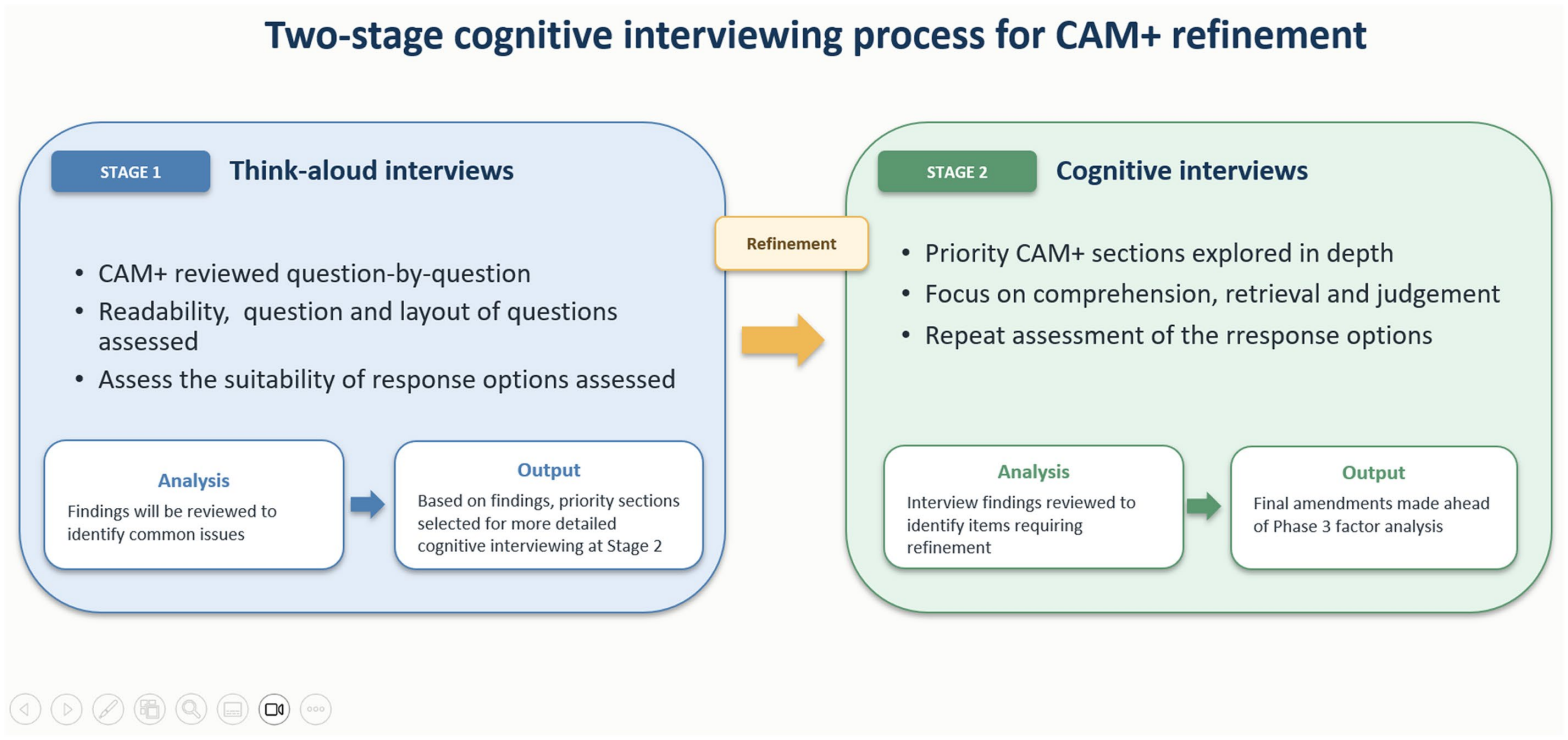
Cognitive interview stages.

This phase will be conducted in two stages to inform minor initial amendments and identify CAM + sections of priority for further cognitive interviewing, see Figure 1 that outlines these stages. The first stage will use a ‘Think Aloud’ method to generate reactive feedback on the wording of questions, responses and images used in the measure. Findings from this initial phase will subsequently inform areas of focus for Stage 2. It is anticipated n = 5 individuals will participate in this first stage.

In the second stage, the interviews will be informed by a semi-structured interview guide based on a four-stage model of cognitive interviewing (46). This process involves asking the individual to consider four aspects of each question:

1. Comprehension: explore comprehension of key terms within the question and the question as a whole.
2. Retrieval: establish whether respondents can recall the required information and restrict their recall to the reference period specified in the question.
3. Judgement: establish respondents’ strategies when answering. For example, do they use an accurate calculation or take a guess
4. Response: explore whether respondents can map their ‘in mind’ answer onto the answer categories available. Also, check whether any answer categories are missing from the list provided.

Findings from this phase will be shared key stakeholders/users of the measure, primarily within CRUK and academics in the field, for their feedback on any proposed changes.

### Phase 3: Factor analysis

- Factor analysis

Using the revised version of the CAM + from the cognitive interviews, we will conduct factor analysis. This will inform further changes to the measure, such as identifying underlying factors and groups of variables that are highly correlated and can be combined into composite factors condensing items that load onto the same construct. The main aim of factor analysis will be to remove redundant variables or sections from the new CAM, where possible, or to identify whether some questionnaire items can be grouped into clusters representing different dimensions of the construct under study. This will ensure the measure is not unnecessarily long and therefore improve completion rates.

The Factor Analysis will be divided into two phases, which will serve different purposes: (1) Exploratory Factor Analysis (EFA), and (2) Confirmatory Factor Analysis (CFA). Performing EFA first helps identify the underlying factor structure of a measure without imposing a predefined model, while CFA then tests and verifies this structure in a separate, independent sample, ensuring validity and reliability (47, 48). This sequential approach improves measurement accuracy by reducing bias, refining identified constructs, and confirming theoretical assumptions (47, 48).

More specifically, EFA will explore and identify potential underlying factors or dimensions within the collected data (first sample). The results will provide insights into how the observed variables cluster together and what underlying constructs may be responsible for these patterns (47). We will then run CFA to test whether the proposed factor structure, based on theory and evidence, fits the data well (second sample). We will evaluate the goodness-of-fit of the hypothesised model to the data and assess whether the proposed factors adequately explain the observed correlations among variables within the new CAM+. Using two separate samples to run EFA, followed by CFA, will reduce potential bias and provide a more rigorous test of the hypothesised factor structure. The comparison of EFA and CFA will help us in validating the findings. If the results of EFA and CFA are consistent, it will ensure greater confidence in the identified factor structure. On the other hand, if the results differ between EFA and CFA, we will engage in further investigation into the reasons behind the discrepancies, such as potential methodological issues or the need to refine the factors or theoretical model.

### Phase 4: Construct validity, reliability and responsiveness

In Phase 4 we will assess construct validity, by comparing responses of cancer experts *vs.* non-cancer experts; reliability, using a test–retest approach; and responsiveness, examining sensitivity to change, following a brief intervention.

- *Construct validity*

We will measure construct validity of the CAM + by comparing responses of cancer experts and experts in other health fields. The aim is to determine whether scores between these two groups differ in ways we would expect, such as higher recognition of cancer signs/symptoms among experts. Construct validity will be assessed by comparing responses from cancer experts based in CRUK and non-cancer experts that work in Third Sector organisations not related to cancer, such as Dementia UK, British Heart Foundation, etc.

- *Test–retest*

The aim of the test–retest reliability is to establish whether the CAM + results are stable across two occasions of measurement: test and re-test. To establish test–retest reliability, relevant items of the newly updated version of CAM + will be piloted with approximately 200 adults (18 + year-olds) living in the UK. They will complete the CAM + online on two occasions, 2 weeks apart (“test–retest”). A two-week interval was selected in line with previous psychometric test–retest studies (4, 49, 50), as it is considered sufficiently short to minimise meaningful change in the underlying construct while reducing immediate recall of previous responses. Participants will be recruited using a national panel managed by a leading market agency, ensuring a broadly nationally representative sample based on age, gender, ethnicity and socio-economic position with sufficient diversity in characteristics.

We will exclude some questions that focus on attitudes and behaviours likely to change in the 2 weeks measurement interval, due to external or internal factors because they are not relevant for assessing the stability of the measure. These include: ‘Thinking about last week, how many units of alcohol did you drink?’ or items measuring awareness of screening programmes which participants would have been informed of while completing the measure at time 1, or open-ended text format questions offered as ‘other’ options in the barriers and prompts to help-seeking items, which therefore do not lend themselves to the computation of psychometric coefficients.

- *Sensitivity to change analysis*

Considering that CAM results have been used to evaluate local and national health campaigns (3, 19), we wanted to evaluate whether the revised CAM + can detect meaningful changes in cancer awareness, attitudes and behaviour over time. The primary aim of this responsiveness assessment is psychometric evaluation of sensitivity to change, rather than evaluation of intervention effectiveness. However, once revalidated, the CAM + may subsequently be used to evaluate the impact of awareness-raising campaigns and other interventions designed to improve cancer awareness, attitudes, and help-seeking behaviours (51).

We will design and deliver a brief intervention to assess sensitivity to change, using the CAM + before and after the intervention. We will start by conducting a scoping review to identify which Behavioural Change Techniques (BCTs) have been successfully used in brief interventions to improve cancer awareness, attitudes and behaviours. For example, we will look for interventions focused on encouraging help seeking behaviour when people notice a symptom they have just been educated about; or screening uptake following a nurse-led timely screening attendance workshop. The search will be limited to interventions delivered within the last decade (2014–2024), and papers published in English only, but not limited to the UK context.

We will focus only on awareness, attitudes and behaviours that may be amenable to change via an intervention. Specifically, we will identify brief interventions which have been used to:

- Improve awareness of risk factors for cancer
- Improve awareness of potential signs/symptoms of cancer
- Improve awareness AND uptake of cancer screening (cervical, bowel, breast), and
- Improve attitudes towards cancer screening

Based on these results we will develop and deliver an intervention, which will be a one-time event, run for approximately 2–3 h. The content and mode of delivery will be determined based on evidence generated from similar brief interventions. We will use an appropriate range of educational resources chosen to reflect successful previous interventions as per results of the scoping reviews.

### Overview of planned studies

**Figure 2:**
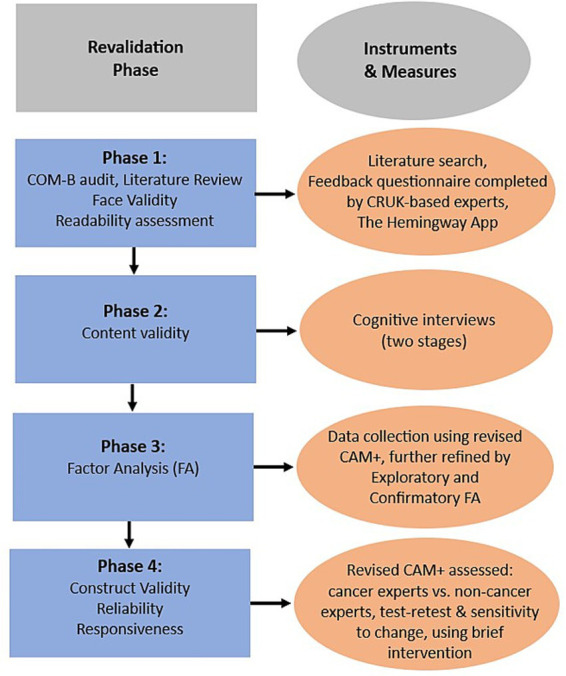
Flow chart of the planned CAM + revalidation studies.

Figure 2 shows the Flow chart of the planned CAM + revalidation studies, including research approach/design, and instruments and measures used within each phase.

### Participants

For the activities involving primary data collection (face validity, cognitive interviewing, FA, construct validity, reliability, and responsiveness) the criteria for recruitment will be:

#### Inclusion criteria

- Adults aged 18+.
- Able to give informed consent.
- Currently reside in the UK.
- Face Validity study only – “cancer expert”: Eligible participants included individuals working in cancer-related clinical practice, cancer epidemiology, behavioural science, cancer policy, cancer awareness campaigns, or cancer research roles, with demonstrable professional expertise relevant to cancer awareness, symptom appraisal, screening, or early diagnosis. For example, CRUK staff with relevant expertise, external stakeholders and academics with expertise in cancer awareness, prevention, behavioural science, screening, and early diagnosis.

### Recruitment and data collection

Recruitment processes for primary data collection are described below. All numbers are estimations.

Face Validity (*N* = 50): CRUK will coordinate recruitment by circulating the opportunity to colleagues through internal networks and targeted invitations to key informants, i.e., experts in cancer research.

Cognitive Interviews (*N* = 20: Cognitive Interviews (*N* = 5, Phase 1; *N* = 15) Phase 2: An external research agency commissioned by CRUK will purposively recruit individuals on behalf of CRUK and CHSS. We will recruit a diverse sample, including respondents who may experience difficulties reading and understanding the CAM + e.g. lower literacy, people from ethnic minority backgrounds, lower SEP groups.

The agency will circulate the Participant Information Sheet (PIS) and an ‘Expression of Interest’ form that interested individuals will complete and return to a named CHSS researcher.

For Factor Analysis (FA), a market research agency will be commissioned by CRUK to recruit two nationally representative samples (of 1,500 each, one for the EFA and one for the CFA). The sample will be recruited to be nationally representative for age, gender, ethnicity, SEP and region.

Test–retest (*N* = 200): An external research agency will purposively recruit individuals on behalf of CRUK and CHSS using their existing panel. Participants will be diverse enough to aim for representativeness, although this will not be monitored precisely.

Sensitivity analysis (*N* = 300). An external research agency will purposively recruit individuals on behalf of CRUK and CHSS using their existing panel.

Construct Validity (*N* = 100, approximately 50 cancer experts and 50 non-cancer experts). CHSS and CRUK will use professional contacts and networks to offer the opportunity to participate to cancer and non-cancer experts. Interested individuals will complete an online Expression of Interest form to include contact details and whether they identify as a cancer expert or non-expert. The CHSS research team will then contact individuals with a consent form, PIS and the link to the revised CAM + to complete online.

The important topics for inclusion by CRUK are listed below:

- Recognition of potential cancer symptoms
- Recognition of cancer risk factors
- Barriers to seeking medical help
- Prompts to seeking medical help
- Enablers of attending hospital tests
- Barriers to uptake of cervical cancer screening
- Barriers to uptake of bowel cancer screening
- Barriers to uptake of breast cancer screening

### Instruments

#### Questionnaire

The most recent CAM + questionnaire (Appendix I) was run in September 2023. It incorporates questions regarding cancer symptom awareness, attitudes, and behaviour in relation to cancer prevention, early diagnosis and screening.

#### Open ended questions in cognitive interviews

The interview questions are shown in Appendix II and will last between 40 and 60 min.

### Payment for participation

The recruitment agency will offer payment or credits to take part in the cognitive interviews, factor analysis, sensitivity to change and test–retest studies.

### Analysis plan

All quantitative data will be analysed using Stata v.18 (52).

- COM-B audit

Three researchers will independently map items to COM-B domains using a predefined coding framework. Coding discrepancies will be discussed and resolved through consensus. Given the interpretive and theory-informed nature of COM-B framework mapping, consensus-based coding procedures were considered more appropriate (53, 54), than formal inter-rater reliability statistics (55).

- Cognitive interviews

We will interview approximately 20 participants. Data will be transcribed verbatim and analysed using NVivo software. To analyse data, we will reduce and synthesise the data using five incremental steps:

1. Conducting interviews.
2. Producing summaries.
3. Comparing across respondents.
4. Comparing across subgroups of respondents.
5. Reaching conclusions.

As each step is completed, data are reduced such that meaningful content is systematically extracted to produce a summary that details a question’s performance. In detailing a question’s performance, it is possible to understand the ways in which various groups of respondents interpret a question, the processes that respondents utilise to formulate a response as well as any difficulties that respondents might experience when attempting to answer the question (56).

- Factor analysis

First, we will use the Kaiser-Meyer-Olkin (KMO) measure of sampling adequacy to understand whether variables in each sub-scale have enough in common to warrant a factor analysis. Second, we will calculate the Cronbach’s alpha, to assess whether the questions used to assess different sub-scales within CAM + can produce a reliable scale, and whether they actually measure the same construct (internal consistency of a scale). Third, we will perform the Bartlett’s test of sphericity to tests the hypothesis that the correlation matrix is an identity matrix. This will inform us whether we can reject the null hypothesis that the correlation matrix is an identity matrix, suggesting that there are significant correlations among the variables and that factor analysis is appropriate. Fourth, for selection of latent factors we will use the Kaiser-Guttman criterion, the most commonly used criterion, which suggests to retain only factors accounting for the variance of more than one variable, i.e., to drop all factors with eigenvalues under 1 (57). Finally, we will cross-validate this with the visual inspection and scree test for each subscale.

- Construct validity: cancer experts vs. non-experts

The differences between answers given by cancer experts vs. non-experts will be analysed using the appropriate parametric or non-parametric tests, depending on the level of measurement, such as mean scores, paired t-tests, Fisher’s Exact Test, etc.

- Test–retest

Intraclass correlations (95% CI) will be used to assess test–retest agreement over a period of 2 weeks for the new CAM + questionnaire, including, e.g., scales of symptom awareness, barriers to help seeking. We will also use Cronbach’s alpha.

- Sensitivity to change

The differences between answers given by participants at the two repeated time points will be analysed using the appropriate parametric or non-parametric tests, depending on the level of measurement, such as mean scores, paired *t*-tests, McNemar’s test to compare pre- and post-intervention CAM + scores.

### Patient and public involvement statement

The re-validation work will be supported by a lived experience group of 4 people, patients or members of the public, who have either had cancer themselves or cared for a significant other with cancer. This group will be invited to collaborate with the research team on all elements of the re-validation work, including involvement in the design of studies, conducting, reporting, and research dissemination plans. The exact nature of this work will be decided with the group, but it is expected to include tasks such as reviewing of participant recruitment materials, co-designing of cognitive interview guides, and informing dissemination pathways.

### Timeline summary

This protocol describes a multi-year revalidation programme initiated in 2023, including protocol development, stakeholder consultation, ethics approval, and preparatory methodological work. Data collection and psychometric testing are being conducted between 2023 and 2026, with some validation activities, final analyses and dissemination continuing into 2027. The planned study timeline is provided in the Supplementary materials. The purpose of publishing this protocol at this stage is to ensure methodological transparency and provide a public record of the planned validation procedures prior to completion of all study phases. It is also anticipated that the protocol’s comprehensive methodology may inform future validation and revalidation studies of cancer awareness measures and related public health instruments.

### Limitations and potential pitfalls

This revalidation study has several potential limitations. First, some phases rely on purposive and professional network-based recruitment, particularly the construct validity and face validity studies. This may introduce volunteer or affiliation bias and over-represent highly engaged participants with greater cancer-related knowledge than the wider population. However, these phases are intended primarily as known-groups psychometric comparisons, e.g., to assess whether CAM + discriminates between groups expected to differ in cancer awareness, rather than representative epidemiological sampling. Second, the sensitivity to change study uses a repeated pre-post measurement within the same, without a control group. This is commonly used in responsiveness testing to determine whether a measure can detect expected directional change following exposure to a brief educational intervention (58, 59). Nevertheless, because the study is not designed as a controlled intervention trial, observed changes cannot be attributed exclusively to the intervention and may partly reflect testing or practice effects, regression to the mean, or spontaneous change due to natural variation over time.

Third, as part of our reliability testing, we used a two-week test–retest interval, consistent with psychometric recommendations balancing recall effects against the possibility of genuine change in the construct being measured. Nevertheless, some true change in awareness or attitudes may still occur during this interval, particularly for constructs influenced by ongoing exposure to health information. Fourth, repeated completion of CAM + during the test–retest phase may itself increase participants’ awareness of cancer symptoms or help-seeking behaviours, potentially influencing responses at the second measurement point. To minimise this sensitisation effect, items most susceptible to immediate learning or behavioral fluctuation will be excluded from test–retest analyses where appropriate.

Fifth, while nationally representative sampling strategies will be used for large quantitative phases, some under-served populations may remain difficult to recruit or under-represented in online survey panels, including individuals with low digital literacy, limited English proficiency, or unstable housing circumstances.

Finally, although the protocol incorporates multiple approaches to establishing validity and reliability in accordance with COSMIN guidance, future validation work may still be required for translated versions of CAM+, site-specific CAMs, and populations outside the UK context.
