# Supplementary material for: Re-validation of the Cancer Research UK Cancer Awareness Measure ‘plus’ (CAM+): a study protocol
Source: Front Public Health. 2026 Jun 24;14:1841597. doi: 10.3389/fpubh.2026.1841597 (PMC13341668; doi:10.3389/fpubh.2026.1841597)
Supplement: Supplementary file 2 [file Data_Sheet_1.pdf]

YouGov

CRUK_CAM_September_2023

Question type: ***Text***

Welcome to this survey about health attitudes and behaviours conducted by Cancer Research UK. We will be using the results to inform what we should be doing now and in the future. Anonymised results will be shared with public health bodies across the UK, NHS organisations and charities, and the general public. It is our intention to publish the results of this research project in academic journals and present findings at conferences. You will not be identified in any reports, publications or presentations.

The information we collect will be stored safely in a national archive and shared with other researchers so that it can be used for research in the future which is in the public interest. Any information that could identify you will be removed so that no one who may wish to download it in the future will know who you are.

Your participation in this survey is voluntary and you can choose not to answer any question by selecting the ‘Prefer not to say’ option.

Your YouGov Account will be credited with 50 points for completing the survey.

We have tested the survey and found that, on average it takes around 15 minutes to complete. This time may vary depending on factors such as your Internet connection speed and the answers you give.

Please click the forward button below to continue.

Question type: ***Text***

To start, we would like to ask a few questions about your day-to-day life.

Question type: ***PDL***

Smoker status (smoker/non-smoker)

[smoker] {single varlabel = "Smoker/non-smoker"} Which, if any, of the following statements BEST applies to you, when it comes to smoking?

<1> I smoke every day
<2> I smoke but I don't smoke every day
<3> I used to smoke but I have given up now

<4> I have never smoked
<99> Prefer not to say

Base: All
Question type: ***Dropdown***

[Q2] Thinking about last week, on how many days did you take part in physical activity for more than 30 minutes? (by physical activity we mean anything that leaves you warm and slightly out of breath, such as brisk walking, gardening, dancing or doing housework)

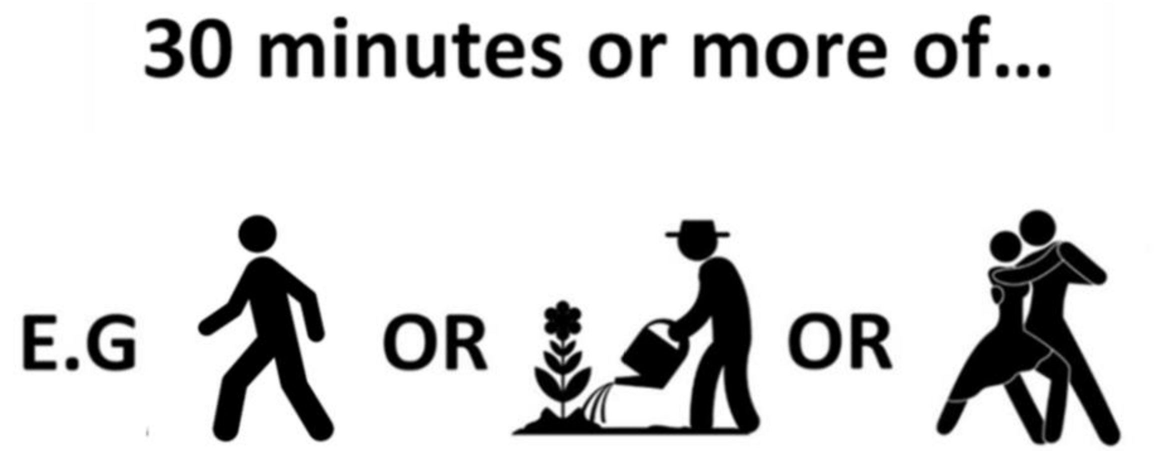

Please select one answer.

<1> 0
<2> 1
<3> 2
<4> 3
<5> 4
<6> 5
<7> 6
<8> Every day
<9> Don't know
<10> Prefer not to say

Base: All
Question type: ***Dropdown***

[Q3] Thinking about last week, how many units of alcohol did you drink?

(A unit of alcohol is one small measure of spirits, half a pint of lager (3-4% strength) or half a small glass (175ml) of wine (12% strength))

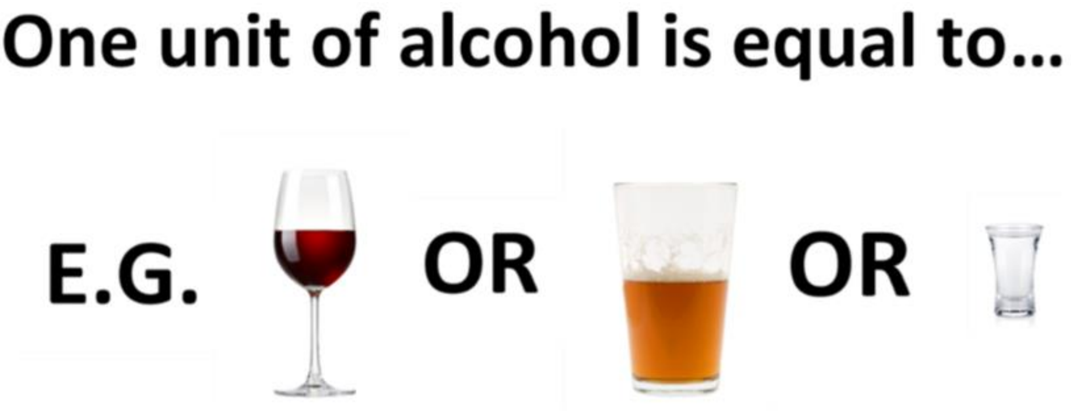

Please select one answer.

<1> 0
<2> 1
<3> 2
<4> 3
<5> 4
....
<101> 100
<102> Prefer not to say
<103> Don’t know

Base: All
Question type: Grid
#row order: randomize

[Q6] Are you currently trying to do any of the following?

Please select one answer per statement.

-[Q6_1 if smoker=1,2] Reduce the amount you smoke
-[Q6_2 if smoker=1,2] Stop smoking completely
-[Q6_3] Decrease the amount of processed meat you eat (e.g. bacon, ham, salami, corned beef, sausages)
-[Q6_4] Increase the amount of physical activity you do
-[Q6_5] Reduce the amount of alcohol you drink
-[Q6_6] Lose weight
-[Q6_7] Eat less food and drink high in fat, salt and sugar (e.g. chocolate, crisps, chips, sugary drinks like Coca Cola)

<1> Yes
<2> No
<3> Maybe

<4> Prefer not to say
<5> This is not applicable to me

Question type: ***Text***

Thank you for your answers so far. The following questions are about your experience of health symptoms in the last 6 months.

Base: All
Question type: ***Grid***
#SPD Category: health

[Q8] In the last 6 months, have you experienced any of the following health symptoms?
Please select one answer per statement.

-[Q8_1] Unexplained weight loss
-[Q8_2] An unexplained lump or swelling
-[Q8_3] A change in the appearance of a mole
-[Q8_4] A persistent change in bowel habits (persistent means doesn't go away) e.g. needing to poo more often, looser or firmer poos
-[Q8_5] A persistent change in bladder habits (persistent means doesn't go away) e.g. needing to wee more often
-[Q8_6] A persistent unexplained pain (persistent means doesn't go away)
-[Q8_7] A persistent difficulty swallowing (persistent means doesn't go away)
-[Q8_8] A persistent cough (persistent means doesn't go away)
-[Q8_9] Unexplained bleeding
-[Q8_10] A sore that does not heal
-[Q8_11] Persistent hoarseness (persistent means doesn't go away)
-[Q8_12] Coughing up blood
-[Q8_13] Feeling tired all the time
-[Q8_14] A change in an existing cough
-[Q8_15] Shortness of breath
-[Q8_16] An ulcer in the mouth that doesn't heal
-[Q8_17] Red or white patches in your mouth

<1> Yes
<2> No
<3> Prefer not to say

#Module display logic:
If [Q8] - Unexplained weight loss, Yes is selected [if Q8_1 == 1]

Question type: ***Text***

You said that you have experienced **unexplained weight loss** in the last 6 months. We would now like to ask you a few more questions about this.

Base: All experiencing each symptom
*Question type: **Single***
#SPD Category: health

[Q12_1] Approximately when did you first notice this symptom? Please give your best guess.
Please select one answer.

Response Option List: Q12_list

[[Q12_list]]
<1> Less than 1 week ago
<2> Less than 2 weeks ago
<3> Less than 1 month ago
<4> Less than 6 weeks ago
<5> Less than 3 months ago
<6> Less than 6 months ago
<7> 6 months ago or longer
<8> Prefer not to say

Base: All experiencing each symptom
*Question type: **Single***
#SPD Category: health

[Q13_1] You said that you have experienced **unexplained weight loss** in the last 6 months. We would now like to ask you a few more questions about this.

How concerned have you been that this symptom might be serious?
Please select one answer.

Response Option List: Q13_list

[[Q13_list]]
<1> Not at all concerned
<2> A little bit concerned
<3> Moderately concerned
<4> Quite a bit concerned
<5> Extremely concerned
<6> Prefer not to say

Base: All experiencing each symptom
*Question type: **Multiple***
#row order: randomize
#SPD Category: health

[Q14_1] You said that you have experienced **unexplained weight loss** in the last 6 months. We would now like to ask you a few more questions about this.

What do you think caused this symptom?
Please select all that apply.

[[Q14_1_list]]

<1> A NEW physical health problem (e.g. a new condition such as a new infection, heart problem or allergic reaction)

<2> An EXISTING physical health problem that you already know you have (e.g. a symptom of having diabetes or high blood pressure)

<3> Medication or vaccination side effects (e.g. a side effect of medication for high blood pressure or diabetes, or a side effect of vaccination)

<4> Psychological health problem (e.g. mental health problems such as depression, stress or anxiety)

<5> External and lifestyle factors (e.g. weather changes, lack of sleep, changes in diet and exercise)

<6> Cancer (e.g. possible cancerous symptom or cancer diagnosis)

<7> COVID-19 infection (physical e.g. symptoms that could be COVID-19 infection such as a high temperature or change in smell)

<7a> Long COVID (physical e.g. long-term symptoms that last weeks or months after COVID-19 infection has gone such as extreme tiredness or changes to sense of smell or taste)

<8> Psychological effects of the COVID-19 pandemic (e.g. mental health problems related to COVID-19, such as boredom, loneliness, financial pressures and COVID-related stress/worry)

<9 fixed xor>	I don't know/Not sure
<10 fixed xor>	Other (open [Q14_1_10open]) [open]
<11 fixed xor>	Prefer not to say

Base: All experiencing each symptom
*Question type: **Single***
#SPD Category: health

[Q15_1] You said that you have experienced **unexplained weight loss** in the last 6 months. We would now like to ask you a few more questions about this.

How long after you first noticed the symptom did you contact the GP (doctor's surgery) about

it? If you are unsure, please give your best guess.
Please select one answer.

Response Option List: Q15_list

[[Q15_list]]
<1> Did not contact the GP
<2> Not contacted the GP yet, but plan to
<3> Within 1 week of noticing the symptom
<4> Within 2 weeks of noticing the symptom
<5> Within 3 weeks of noticing the symptom
<6> Within 1 month of noticing the symptom
<7> Within 6 weeks of noticing the symptom
<8> Within 3 months of noticing the symptom
<9> Within 6 months of noticing the symptom
<10> Prefer not to say

Base: All who made an appointment
*Question type: **Single***
#SPD Category: health
#Question display logic:
if Q15_1 in [3,4,5,6,7,8,9]

[Q15a_1] You said that you contacted your doctor after experiencing **unexplained weight loss** in the last 6 months. How long after first contacting the GP (doctor's surgery) did the appointment take place?

This may have been an appointment that was face-to-face or remote, such as over the phone, by video call or online messaging. This also includes if a medical/health professional called you back after completing an online/e-consultation form.

If you are unsure, please give your best guess.

<1>	On the same day
<2>	On the next day
<3>	A few days later
<4>	A week later
<5>	Two weeks later
<6>	Three weeks later
<7>	Four or more weeks later
<8>	I have not had an appointment yet
<9>	Prefer not to say

Base: All who made an appointment
*Question type: **Single***
#SPD Category: health
#Question display logic:
if Q15_1 in [3,4,5,6,7,8,9] and Q15a_1 != 8

[Q15b_1] You said that you have discussed your **unexplained weight loss** with your doctor (GP).

Did you continue to experience the symptom after you first discussed it with your doctor (GP)?

<1>	Yes, I continued to experience the symptom and it was the same as before
<2>	Yes, I continued to experience the symptom, but it got better
<3>	Yes, I continued to experience the symptom and it got worse
<4>	No, the symptom went away by itself
<5>	No, the symptom went away after taking medication
<95>	Other (open [Q15b_1_open]) [open] Please specify
<99>	Prefer not to say

Base: all who continued to experience the symptom after discussing it with their doctor
*Question type: **Single***
#SPD Category: health
#Question display logic:
If [Q15b_1] - Yes, I continued to experience the symptom and it was the same as before or Yes, I continued to experience the symptom, but it got better or Yes, I continued to experience the symptom and it got worse or Other, is selected [if Q15b_1 in [1,2,3,95]]

[Q15c_1] You said you were still experiencing **unexplained weight loss** after discussing it with your doctor (GP). Did you contact your doctor again after noticing that you were still experiencing the symptom?

<1>	Yes
<2>	No, but I plan to
<3>	No, and I don’t plan to
<99>	Not sure
<100>	Prefer not to say

Base: all who contacted their doctor again
*Question type: **Single***
#SPD Category: health
#Question display logic:
If [Q15c_1] - Yes is selected [if Q15c_1 == 1]

[Q15d_1] You said that you contacted your doctor (GP) again after noticing that you were still experiencing **unexplained weight loss** in the last 6 months.

How long after you noticed that you were still experiencing the symptom did you contact your doctor (GP) again? If you are unsure, please give your best guess.

<1>	Within 1 week
<2>	Within 2 weeks
<3>	Within 3 weeks
<4>	Within 1 month

<5>	Within 6 weeks
<6>	Within 3 months
<7>	Within 6 months
<99>	Prefer not to say

Base: All experiencing each symptom
*Question type: **Single***
#SPD Category: health

[Q15e_1] You said that you have experienced **unexplained weight loss** in the last 6 months. Have you had any tests or investigations to find out what is causing this symptom?

<1>	Yes
<2>	No, but I am waiting for a test
<3>	No, and I am not waiting for a test
<98>	Not sure / don't know
<99>	Prefer not to say

Base: all who had a test
*Question type: **Single***
#SPD Category: health
#Question display logic:
If [Q15e_1] - Yes is selected [if Q15e_1 == 1]

[Q15f_1] You said that you had a test or investigation for **unexplained weight loss** in the last 6 months. What were the results of your tests?

<1>	The results suggested there was nothing to worry about
<2>	The results meant I needed more tests (to investigate further)
<3>	The results meant I needed to be monitored and retested again later
<4>	The results led to a diagnosis
<5>	I have not received my test results yet
<6>	I don't remember what the test results were
<95>	Other (open [Q15f_1_open]) [open] Please specify
<99>	Prefer not to say

Base: all who had a test
*Question type: **Single***
#SPD Category: health
#Question display logic:
If [Q15f_1] - The results suggested there was nothing to worry about is selected [if Q15f_1 == 1]

[Q15g_1] Did you continue to experience the symptom after your test result said there was nothing to worry about?
Please select one answer.

<1> Yes, I continued to experience the symptom and it was the same as before
<2> Yes, I continued to experience the symptom, but it got better
<3> Yes, I continued to experience the symptom and it got worse
<4> No, the symptom went away by itself
<5> No, the symptom went away after taking medication
<95> Other (open [Q15g_1_open]) [open] Please specify
<99> Prefer not to say

---

*Question type: **Single***
#Question display logic:
If [Q15g_1] - Yes, I continued to experience the symptom and it was the same as before or Yes, I continued to experience the symptom, but it got better or Yes, I continued to experience the symptom and it got worse or Other, is selected [if Q15g_1 in [1,2,3,95]]

[Q15h_1] You said you were still experiencing **unexplained weight loss** after receiving your test results. Did you contact your doctor again after noticing that you were still experiencing the symptom?

<1> Yes
<2> No, but I plan to
<3> No, and I don't plan to
<99> Not sure
<100> Prefer not to say

---

*Question type: **Single***
#Question display logic:
If [Q15h_1] - Yes is selected [if Q15h_1 == 1]

[Q15i_1] You said that you contacted your doctor (GP) again after noticing that you were still experiencing **unexplained weight loss** after receiving your test results.

How long after you noticed that you were still experiencing the symptom did you contact your doctor (GP) again? If you are unsure, please give your best guess.

<1> Within 1 week
<2> Within 2 weeks
<3> Within 3 weeks
<4> Within 1 month
<5> Within 6 weeks
<6> Within 3 months
<7> Within 6 months
<99> Prefer not to say

#Module display logic:
If [Q8] - An unexplained lump or swelling, Yes is selected [if Q8_2 == 1]

Question type: ***Text***

You said that you have experienced **an unexplained lump or swelling** in the last 6 months. We would now like to ask you a few more questions about this.

Base: All experiencing each symptom
Question type: ***Single***
#SPD Category: health

[Q9] Where in the body was the lump or swelling?
Please select one answer.

<1>	Breast
<2>	Neck
<3>	Armpit
<4>	Groin
<5>	Chest
<6>	Testicle
<7>	Abdomen (tummy)
<8 fixed>	Other (Please specify)
<9 fixed>	Prefer not to say

Base: All experiencing each symptom
Question type: ***Single***
#SPD Category: health

[Q12_2] You said that you have experienced **an unexplained lump or swelling** in the last 6 months. We would now like to ask you a few more questions about this.

Approximately when did you first notice this symptom? Please give your best guess.
Please select one answer.

Response Option List: Q12_list

Base: All experiencing each symptom
Question type: ***Single***
#SPD Category: health

[Q13_2] You said that you have experienced **an unexplained lump or swelling** in the last 6 months. We would now like to ask you a few more questions about this.

How concerned have you been that this symptom might be serious?
Please select one answer.

Response Option List: Q13_list

Base: All experiencing each symptom
*Question type: **Multiple***
#row order: randomize
#SPD Category: health

[Q14_2] You said that you have experienced **an unexplained lump or swelling** in the last 6 months. We would now like to ask you a few more questions about this.

What do you think caused this symptom?
Please select all that apply.

Response Option List: Q14_list

Base: All experiencing each symptom
*Question type: **Single***
#SPD Category: health

[Q15_2] You said that you have experienced **an unexplained lump or swelling** in the last 6 months. We would now like to ask you a few more questions about this.

How long after you first noticed the symptom did you contact the GP (doctor's surgery) about it? If you are unsure, please give your best guess.
Please select one answer.

Response Option List: Q15_list

Base: All who made an appointment
*Question type: **Single***
#SPD Category: health
#Question display logic:
if Q15_2 in [3,4,5,6,7,8,9]

[Q15a_2] You said that you contacted your doctor after experiencing **an unexplained lump or swelling** in the last 6 months. How long after first contacting the GP (doctor's surgery) did the appointment take place?

This may have been an appointment that was face-to-face or remote, such as over the phone, by video call or online messaging. This also includes if a medical/health professional called you back after completing an online/e-consultation form.

>If you are unsure, please give your best guess.

<1>	On the same day
<2>	On the next day
<3>	A few days later

<4>	A week later
<5>	Two weeks later
<6>	Three weeks later
<7>	Four or more weeks later
<8>	I have not had an appointment yet
<9>	Prefer not to say

Base: All who made an appointment
*Question type: **Single***
#SPD Category: health
#Question display logic:
if Q15_2 in [3,4,5,6,7,8,9] and Q15a_2 != 8

[Q15b_2] You said that you have discussed the **unexplained lump or swelling** with your doctor (GP). Did you continue to experience the symptom after you first discussed it with your doctor (GP)?

<1>	Yes, I continued to experience the symptom and it was the same as before
<2>	Yes, I continued to experience the symptom, but it got better
<3>	Yes, I continued to experience the symptom and it got worse
<4>	No, the symptom went away by itself
<5>	No, the symptom went away after taking medication
<95>	Other (open [Q15b_2_open]) [open] Please specify
<99>	Prefer not to say

Base: all who continued to experience the symptom after discussing it with their doctor
*Question type: **Single***
#SPD Category: health
#Question display logic:
If [Q15b_2] - Yes, I continued to experience the symptom and it was the same as before or Yes, I continued to experience the symptom, but it got better or Yes, I continued to experience the symptom and it got worse or Other, is selected [if Q15b_2 in [1,2,3,95]]

[Q15c_2] You said you were still experiencing **an unexplained lump or swelling** after discussing it with your doctor (GP). Did you contact your doctor again after noticing that you were still experiencing the symptom?

<1>	Yes
<2>	No, but I plan to
<3>	No, and I don’t plan to
<99>	Not sure
<100>	Prefer not to say

Base: all who contacted their doctor again
*Question type: **Single***

#SPD Category: health
#Question display logic:
If [Q15c_2] - Yes is selected [if Q15c_2 == 1]

[Q15d_2] You said that you contacted your doctor (GP) again after noticing that you were still experiencing **an unexplained lump or swelling** in the last 6 months. How long after you noticed that you were still experiencing the symptom did you contact your doctor (GP) again? If you are unsure, please give your best guess.

<1>	Within 1 week
<2>	Within 2 weeks
<3>	Within 3 weeks
<4>	Within 1 month
<5>	Within 6 weeks
<6>	Within 3 months
<7>	Within 6 months
<99>	Prefer not to say

Base: all who contacted their doctor again
Question type: ***Single***
#SPD Category: health

[Q15e_2] You said that you have experienced **an unexplained lump or swelling** in the last 6 months. Have you had any tests or investigations to find out what is causing this symptom?

<1>	Yes
<2>	No, but I am waiting for a test
<3>	No, and I am not waiting for a test
<98>	Not sure / don’t know
<99>	Prefer not to say

Base: all who had a test
Question type: ***Single***
#SPD Category: health
#Question display logic:
If [Q15e_2] - Yes is selected [if Q15e_2 == 1]

[Q15f_2] You said that you had a test or investigation for **an unexplained lump or swelling** in the last 6 months. What were the results of your tests?

<1>	The results suggested there was nothing to worry about
<2>	The results meant I needed more tests (to investigate further)
<3>	The results meant I needed to be monitored and retested again later
<4>	The results led to a diagnosis
<5>	I have not received my test results yet
<6>	I don’t remember what the test results were
<95>	Other (open [Q15f_2_open]) [open] Please specify
<99>	Prefer not to say

Base: all who had a test
*Question type: **Single***
#SPD Category: health
#Question display logic:
If [Q15f_2] - The results suggested there was nothing to worry about is selected [if Q15f_2 == 1]

[Q15g_2] Did you continue to experience the symptom after your test result said there was nothing to worry about?
Please select one answer.

<1>	Yes, I continued to experience the symptom and it was the same as before
<2>	Yes, I continued to experience the symptom, but it got better
<3>	Yes, I continued to experience the symptom and it got worse
<4>	No, the symptom went away by itself
<5>	No, the symptom went away after taking medication
<95>	Other (open [Q15g_2_open]) [open] Please specify
<99>	Prefer not to say

---

*Question type: **Single***
#Question display logic:
If [Q15g_2] - Yes, I continued to experience the symptom and it was the same as before or Yes, I continued to experience the symptom, but it got better or Yes, I continued to experience the symptom and it got worse or Other, is selected [if Q15g_2 in [1,2,3,95]]

[Q15h_2] You said you were still experiencing **an unexplained lump or swelling** after receiving your test results. Did you contact your doctor again after noticing that you were still experiencing the symptom?

<1>	Yes
<2>	No, but I plan to
<3>	No, and I don't plan to
<99>	Not sure
<100>	Prefer not to say

---

*Question type: **Single***
#Question display logic:
If [Q15h_2] - Yes is selected [if Q15h_2 == 1]

[Q15i_2] You said that you contacted your doctor (GP) again after noticing that you were still experiencing **an unexplained lump or swelling** after receiving your test results. How long after you noticed that you were still experiencing the symptom did you contact your doctor (GP) again? If you are unsure, please give your best guess.

<1>	Within 1 week

<2>	Within 2 weeks
<3>	Within 3 weeks
<4>	Within 1 month
<5>	Within 6 weeks
<6>	Within 3 months
<7>	Within 6 months
<99>	Prefer not to say

#Module display logic:
If [Q8] - A change in the appearance of a mole, Yes is selected [if Q8_3 == 1]

Question type: ***Text***

You said that you have experienced **a change in the appearance of a mole** in the last 6 months. We would now like to ask you a few more questions about this.

Base: All experiencing each symptom
Question type: ***Single***
#SPD Category: health

[Q12_3] Approximately when did you first notice this symptom? Please give your best guess.
Please select one answer.

Response Option List: Q12_list

Base: All experiencing each symptom
Question type: ***Single***
#SPD Category: health

[Q13_3] You said that you have experienced **a change in the appearance of a mole** in the last 6 months. We would now like to ask you a few more questions about this.

How concerned have you been that this symptom might be serious?
Please select one answer.

Response Option List: Q13_list

Base: All experiencing each symptom
Question type: ***Multiple***
#row order: randomize
#SPD Category: health

[Q14_3] You said that you have experienced **a change in the appearance of a mole** in the last 6 months. We would now like to ask you a few more questions about this.

What do you think caused this symptom?
Please select all that apply.

Response Option List: Q14_list

Base: All experiencing each symptom
Question type: ***Single***
#SPD Category: health

[Q15_3] You said that you have experienced **a change in the appearance of a mole** in the last 6 months. We would now like to ask you a few more questions about this.

How long after you first noticed the symptom did you contact the GP (doctor's surgery) about it? If you are unsure, please give your best guess.
Please select one answer.

Response Option List: Q15_list

Base: All who made an appointment
Question type: ***Single***
#SPD Category: health
#Question display logic:
if Q15_3 in [3,4,5,6,7,8,9]

[Q15a_3] You said that you contacted your doctor after experiencing **a change in the appearance of a mole** in the last 6 months. How long after first contacting the GP (doctor's surgery) did the appointment take place?

This may have been an appointment that was face-to-face or remote, such as over the phone, by video call or online messaging. This also includes if a medical/health professional called you back after completing an online/e-consultation form.

If you are unsure, please give your best guess.

<1>	On the same day
<2>	On the next day
<3>	A few days later
<4>	A week later
<5>	Two weeks later
<6>	Three weeks later

<7>	Four or more weeks later
<8>	I have not had an appointment yet
<9>	Prefer not to say

Base: All who made an appointment
*Question type: **Single***
#SPD Category: health
#Question display logic:
if Q15_3 in [3,4,5,6,7,8,9] and Q15a_3 != 8

[Q15b_3] You said that you have discussed **the change in the appearance of a mole** with your doctor (GP). Did you continue to experience the symptom after you first discussed it with your doctor (GP)?

<1>	Yes, I continued to experience the symptom and it was the same as before
<2>	Yes, I continued to experience the symptom, but it got better
<3>	Yes, I continued to experience the symptom and it got worse
<4>	No, the symptom went away by itself
<5>	No, the symptom went away after taking medication
<95>	Other (open [Q15b_3_open]) [open] Please specify
<99>	Prefer not to say

Base: all who continued to experience the symptom after discussing it with their doctor
*Question type: **Single***
#SPD Category: health
#Question display logic:
If [Q15b_3] - Yes, I continued to experience the symptom and it was the same as before or Yes, I continued to experience the symptom, but it got better or Yes, I continued to experience the symptom and it got worse or Other, is selected [if Q15b_3 in [1,2,3,95]]

[Q15c_3] You said you were still experiencing **a change in the appearance of a mole** after discussing it with your doctor (GP). Did you contact your doctor again after noticing that you were still experiencing the symptom?

<1>	Yes
<2>	No, but I plan to
<3>	No, and I don’t plan to
<99>	Not sure
<100>	Prefer not to say

Base: all who contacted their doctor again
*Question type: **Single***
#SPD Category: health

#Question display logic:
If [Q15c_3] - Yes is selected [if Q15c_3 == 1]

[Q15d_3] You said that you contacted your doctor (GP) again after noticing that you were still experiencing **a change in the appearance of a mole** in the last 6 months.

How long after you noticed that you were still experiencing the symptom did you contact your doctor (GP) again? If you are unsure, please give your best guess.

<1>	Within 1 week
<2>	Within 2 weeks
<3>	Within 3 weeks
<4>	Within 1 month
<5>	Within 6 weeks
<6>	Within 3 months
<7>	Within 6 months
<99>	Prefer not to say

Base: all who contacted their doctor again
Question type: ***Single***
#SPD Category: health

[Q15e_3] You said that you have experienced **a change in the appearance of a mole** in the last 6 months. Have you had any tests or investigations to find out what is causing for this symptom?

<1>	Yes
<2>	No, but I am waiting for a test
<3>	No, and I am not waiting for a test
<98>	Not sure / don’t know
<99>	Prefer not to say

Base: all who had a test
Question type: ***Single***
#SPD Category: health
#Question display logic:
If [Q15e_3] - Yes is selected [if Q15e_3 == 1]

[Q15f_3] You said that you had a test or investigation for **a change in the appearance of a mole** in the last 6 months. What were the results of your tests?

<1>	The results suggested there was nothing to worry about
<2>	The results meant I needed more tests (to investigate further)
<3>	The results meant I needed to be monitored and retested again later
<4>	The results led to a diagnosis
<5>	I have not received my test results yet
<6>	I don’t remember what the test results were
<95>	Other (open [Q15f_3_open]) [open] Please specify
<99>	Prefer not to say

Base: all who had a test
Question type: ***Single***
#SPD Category: health
#Question display logic:
If [Q15f_3] - The results suggested there was nothing to worry about is selected [if Q15f_3 == 1]

[Q15g_3] Did you continue to experience the symptom after your test result said there was nothing to worry about?
Please select one answer.

<1>	Yes, I continued to experience the symptom and it was the same as before
<2>	Yes, I continued to experience the symptom, but it got better
<3>	Yes, I continued to experience the symptom and it got worse
<4>	No, the symptom went away by itself
<5>	No, the symptom went away after taking medication
<95>	Other (open [Q15g_3_open]) [open] Please specify
<99>	Prefer not to say

Question type: ***Single***
#Question display logic:
If [Q15g_3] - Yes, I continued to experience the symptom and it was the same as before or Yes, I continued to experience the symptom, but it got better or Yes, I continued to experience the symptom and it got worse or Other, is selected [if Q15g_3 in [1,2,3,95]]

[Q15h_3] You said you were still experiencing **a change in the appearance of a mole** after receiving your test results. Did you contact your doctor again after noticing that you were still experiencing the symptom?

<1>	Yes
<2>	No, but I plan to
<3>	No, and I don’t plan to
<99>	Not sure
<100>	Prefer not to say

Question type: ***Single***
#Question display logic:
If [Q15h_3] - Yes is selected [if Q15h_3 == 1]

[Q15i_3] You said that you contacted your doctor (GP) again after noticing that you were still experiencing **a change in the appearance of a mole** after receiving your test results. How long after you noticed that you were still experiencing the symptom did you contact your doctor (GP) again? If you are unsure, please give your best guess.

<1>	Within 1 week
<2>	Within 2 weeks
<3>	Within 3 weeks
<4>	Within 1 month
<5>	Within 6 weeks
<6>	Within 3 months
<7>	Within 6 months
<99>	Prefer not to say

#Module display logic:
If [Q8] - A persistent change in bowel habits (persistent means doesn't go away) e.g. needing to poo more often, looser or firmer poos, Yes is selected [if Q8_4 == 1]

Question type: ***Text***

You said that you have experienced a **persistent change in bowel habits** in the last 6 months. We would now like to ask you a few more questions about this.

Base: All experiencing each symptom
Question type: ***Single***
#SPD Category: health

[Q12_4] Approximately when did you first notice this symptom? Please give your best guess.
Please select one answer.

Response Option List: Q12_list

Base: All experiencing each symptom
Question type: ***Single***
#SPD Category: health

[Q13_4] You said that you have experienced a **persistent change in bowel habits** in the last 6 months. We would now like to ask you a few more questions about this.

How concerned have you been that this symptom might be serious?
Please select one answer.

Response Option List: Q13_list

Base: All experiencing each symptom
*Question type: **Multiple***
#row order: randomize
#SPD Category: health

[Q14_4] You said that you have experienced a **persistent change in bowel habits** in the last 6 months. We would now like to ask you a few more questions about this.

What do you think caused this symptom?
Please select all that apply.

Response Option List: Q14_list

Base: All experiencing each symptom
*Question type: **Single***
#SPD Category: health
#Question display logic:
If [Q8] - A persistent change in bowel habits (persistent means doesn't go away) e.g. needing to poo more often, looser or firmer poos, Yes is selected [if Q8_4 == 1]

[Q15_4] You said that you have experienced a **persistent change in bowel habits** in the last 6 months. We would now like to ask you a few more questions about this.

How long after you first noticed the symptom did you contact the GP (doctor's surgery) about it? If you are unsure, please give your best guess.
Please select one answer.

Response Option List: Q15_list

Base: All who made an appointment
*Question type: **Single***
#SPD Category: health
#Question display logic:
if Q15_4 in [3,4,5,6,7,8,9]

[Q15a_4] You said that you contacted your doctor after experiencing a **persistent change in bowel habits** in the last 6 months. How long after first contacting the GP (doctor's surgery) did the appointment take place?

This may have been an appointment that was face-to-face or remote, such as over the phone, by video call or online messaging. This also includes if a medical/health professional called you back after completing an online/e-consultation form.

If you are unsure, please give your best guess.

<1>	On the same day
<2>	On the next day
<3>	A few days later
<4>	A week later
<5>	Two weeks later
<6>	Three weeks later
<7>	Four or more weeks later
<8>	I have not had an appointment yet
<9>	Prefer not to say

Base: All who made an appointment
*Question type: **Single***
#SPD Category: health
#Question display logic:
if Q15_4 in [3,4,5,6,7,8,9] and Q15a_4 != 8

[Q15b_4] You said that you have discussed a **persistent change in bowel habits** with your doctor (GP). Did you continue to experience the symptom after you first discussed it with your doctor (GP)?

<1>	Yes, I continued to experience the symptom and it was the same as before
<2>	Yes, I continued to experience the symptom, but it got better
<3>	Yes, I continued to experience the symptom and it got worse
<4>	No, the symptom went away by itself
<5>	No, the symptom went away after taking medication
<95>	Other (open [Q15b_4_open]) [open] Please specify
<99>	Prefer not to say

Base: all who continued to experience the symptom after discussing it with their doctor
*Question type: **Single***
#SPD Category: health
#Question display logic:
If [Q15b_4] - Yes, I continued to experience the symptom and it was the same as before or Yes, I continued to experience the symptom, but it got better or Yes, I continued to experience the symptom and it got worse or Other, is selected [if Q15b_4 in [1,2,3,95]]

[Q15c_4] You said you were still experiencing a **persistent change in bowel habits** after discussing it with your doctor (GP). Did you contact your doctor again after noticing that you were still experiencing the symptom?

<1>	Yes
<2>	No, but I plan to
<3>	No, and I don’t plan to
<99>	Not sure
<100>	Prefer not to say

Base: all who contacted their doctor again
*Question type: **Single***
#SPD Category: health
#Question display logic:
If [Q15c_4] - Yes is selected [if Q15c_4 == 1]

[Q15d_4] You said that you contacted your doctor (GP) again after noticing that you were still experiencing a **persistent change in bowel habits** in the last 6 months.

How long after you noticed that you were still experiencing the symptom did you contact your doctor (GP) again? If you are unsure, please give your best guess.

<1>	Within 1 week
<2>	Within 2 weeks
<3>	Within 3 weeks
<4>	Within 1 month
<5>	Within 6 weeks
<6>	Within 3 months
<7>	Within 6 months
<99>	Prefer not to say

Base: all who contacted their doctor again
*Question type: **Single***
#SPD Category: health

[Q15e_4] You said that you have experienced a **persistent change in bowel habits** in the last 6 months. Have you had any tests or investigations to find out what is causing this symptom?

<1>	Yes
<2>	No, but I am waiting for a test
<3>	No, and I am not waiting for a test
<98>	Not sure / don't know
<99>	Prefer not to say

Base: all who had a test
*Question type: **Single***
#SPD Category: health
#Question display logic:
If [Q15e_4] - Yes is selected [if Q15e_4 == 1]

[Q15f_4] You said that you had a test or investigation for a **persistent change in bowel habits** in the last 6 months. What were the results of your tests?

<1>	The results suggested there was nothing to worry about
<2>	The results meant I needed more tests (to investigate further)
<3>	The results meant I needed to be monitored and retested again later
<4>	The results led to a diagnosis
<5>	I have not received my test results yet

<6>	I don't remember what the test results were
<95>	Other (open [Q15f_4_open]) [open] Please specify
<99>	Prefer not to say

Base: all who had a test
*Question type: **Single***
#SPD Category: health
#Question display logic:
If [Q15f_4] - The results suggested there was nothing to worry about is selected [if Q15f_4 == 1]

[Q15g_4] Did you continue to experience the symptom after your test result said there was nothing to worry about?
Please select one answer.

<1>	Yes, I continued to experience the symptom and it was the same as before
<2>	Yes, I continued to experience the symptom, but it got better
<3>	Yes, I continued to experience the symptom and it got worse
<4>	No, the symptom went away by itself
<5>	No, the symptom went away after taking medication
<95>	Other (open [Q15g_4_open]) [open] Please specify
<99>	Prefer not to say

---

*Question type: **Single***
#Question display logic:
If [Q15g_4] - Yes, I continued to experience the symptom and it was the same as before or Yes, I continued to experience the symptom, but it got better or Yes, I continued to experience the symptom and it got worse or Other, is selected [if Q15g_4 in [1,2,3,95]]

[Q15h_4] You said you were still experiencing a **persistent change in bowel habits** after receiving your test results. Did you contact your doctor again after noticing that you were still experiencing the symptom?

<1>	Yes
<2>	No, but I plan to
<3>	No, and I don't plan to
<99>	Not sure
<100>	Prefer not to say

---

*Question type: **Single***
#Question display logic:
If [Q15h_4] - Yes is selected [if Q15h_4 == 1]

[Q15i_4] You said that you contacted your doctor (GP) again after noticing that you were still experiencing a **persistent change in bowel habits** after receiving your test results.

How long after you noticed that you were still experiencing the symptom did you contact your doctor (GP) again? If you are unsure, please give your best guess.

<1>	Within 1 week
<2>	Within 2 weeks
<3>	Within 3 weeks
<4>	Within 1 month
<5>	Within 6 weeks
<6>	Within 3 months
<7>	Within 6 months
<99>	Prefer not to say

#Module display logic:
If [Q8] - A persistent change in bladder habits (persistent means doesn't go away) e.g. needing to wee more often, Yes is selected [if Q8_5 == 1]

Question type: ***Text***

You said that you have experienced a **persistent change in bladder habits** in the last 6 months. We would now like to ask you a few more questions about this.

Base: All experiencing each symptom
Question type: ***Single***
#SPD Category: health

[Q12_5] Approximately when did you first notice this symptom? Please give your best guess.
Please select one answer.

Response Option List: Q12_list

Base: All experiencing each symptom
Question type: ***Single***
#SPD Category: health

[Q13_5] You said that you have experienced a **persistent change in bladder habits** in the last 6 months. We would now like to ask you a few more questions about this.

How concerned have you been that this symptom might be serious?
Please select one answer.

Response Option List: Q13_list

Base: All experiencing each symptom
*Question type: **Multiple***
#row order: randomize
#SPD Category: health

[Q14_5] You said that you have experienced a **persistent change in bladder habits** in the last 6 months. We would now like to ask you a few more questions about this.

What do you think caused this symptom?
Please select all that apply.

Response Option List: Q14_list

Base: All experiencing each symptom
*Question type: **Single***
#SPD Category: health

[Q15_5] You said that you have experienced a **persistent change in bladder habits** in the last 6 months. We would now like to ask you a few more questions about this.

How long after you first noticed the symptom did you contact the GP (doctor's surgery) about it? If you are unsure, please give your best guess.
Please select one answer.

Response Option List: Q15_list

Base: All who made an appointment
*Question type: **Single***
#SPD Category: health
#Question display logic:
if Q15_5 in [3,4,5,6,7,8,9]

[Q15a_5] You said that you contacted your doctor after experiencing a **persistent change in bladder habits** in the last 6 months. How long after first contacting the GP (doctor’s surgery) did the appointment take place?

This may have been an appointment that was face-to-face or remote, such as over the phone, by video call or online messaging. This also includes if a medical/health professional called you back after completing an online/e-consultation form.

If you are unsure, please give your best guess.

<1>	On the same day
<2>	On the next day
<3>	A few days later
<4>	A week later
<5>	Two weeks later
<6>	Three weeks later
<7>	Four or more weeks later
<8>	I have not had an appointment yet
<9>	Prefer not to say

Base: All who made an appointment
*Question type: **Single***
#SPD Category: health
#Question display logic:
if Q15_5 in [3,4,5,6,7,8,9] and Q15a_5 != 8

[Q15b_5] You said that you have discussed your **persistent change in bladder habits** with your doctor (GP). Did you continue to experience the symptom after you first discussed it with your doctor (GP)?

<1>	Yes, I continued to experience the symptom and it was the same as before
<2>	Yes, I continued to experience the symptom, but it got better
<3>	Yes, I continued to experience the symptom and it got worse
<4>	No, the symptom went away by itself
<5>	No, the symptom went away after taking medication
<95>	Other (open [Q15b_5_open]) [open] Please specify
<99>	Prefer not to say

Base: all who continued to experience the symptom after discussing it with their doctor
*Question type: **Single***
#SPD Category: health
#Question display logic:
If [Q15b_5] - Yes, I continued to experience the symptom and it was the same as before or Yes, I continued to experience the symptom, but it got better or Yes, I continued to experience the symptom and it got worse or Other, is selected [if Q15b_5 in [1,2,3,95]]

[Q15c_5] You said you were still experiencing a **persistent change in bladder habits** after discussing it with your doctor (GP). Did you contact your doctor again after noticing that you were still experiencing the symptom?

<1>	Yes
<2>	No, but I plan to
<3>	No, and I don’t plan to
<99>	Not sure
<100>	Prefer not to say

Base: all who contacted their doctor again
*Question type: **Single***
#SPD Category: health
#Question display logic:
If [Q15c_5] - Yes is selected [if Q15c_5 == 1]

[Q15d_5] You said that you contacted your doctor (GP) again after noticing that you were still experiencing a **persistent change in bladder habits** in the last 6 months.

How long after you noticed that you were still experiencing the symptom did you contact your doctor (GP) again? If you are unsure, please give your best guess.

<1>	Within 1 week
<2>	Within 2 weeks
<3>	Within 3 weeks
<4>	Within 1 month
<5>	Within 6 weeks
<6>	Within 3 months
<7>	Within 6 months
<99>	Prefer not to say

Base: all who contacted their doctor again
*Question type: **Single***
#SPD Category: health

[Q15e_5] You said that you have experienced a **persistent change in bladder habits** in the last 6 months. Have you had any tests or investigations to find out what is causing this symptom?

<1>	Yes
<2>	No, but I am waiting for a test
<3>	No, and I am not waiting for a test
<98>	Not sure / don’t know
<99>	Prefer not to say

Base: all who had a test
*Question type: **Single***
#SPD Category: health
#Question display logic:
If [Q15e_5] - Yes is selected [if Q15e_5 == 1]

[Q15f_5] You said that you had a test or investigation for a **persistent change in bladder habits** in the last 6 months. What were the results of your tests?

<1>	The results suggested there was nothing to worry about
<2>	The results meant I needed more tests (to investigate further)
<3>	The results meant I needed to be monitored and retested again later
<4>	The results led to a diagnosis
<5>	I have not received my test results yet

<6> I don’t remember what the test results were
<95> Other (open [Q15f_5_open]) [open] Please specify
<99> Prefer not to say

Base: all who had a test
*Question type: **Single***
#SPD Category: health
#Question display logic:
If [Q15f_5] - The results suggested there was nothing to worry about is selected [if Q15f_5 == 1]

[Q15g_5] Did you continue to experience the symptom after your test result said there was nothing to worry about?
Please select one answer.

<1> Yes, I continued to experience the symptom and it was the same as before
<2> Yes, I continued to experience the symptom, but it got better
<3> Yes, I continued to experience the symptom and it got worse
<4> No, the symptom went away by itself
<5> No, the symptom went away after taking medication
<95> Other (open [Q15g_5_open]) [open] Please specify
<99> Prefer not to say

---

*Question type: **Single***
#Question display logic:
If [Q15g_5] - Yes, I continued to experience the symptom and it was the same as before or Yes, I continued to experience the symptom, but it got better or Yes, I continued to experience the symptom and it got worse or Other, is selected [if Q15g_5 in [1,2,3,95]]

[Q15h_5] You said you were still experiencing a **persistent change in bladder habits** after receiving your test results. Did you contact your doctor again after noticing that you were still experiencing the symptom?

<1> Yes
<2> No, but I plan to
<3> No, and I don’t plan to
<99> Not sure
<100> Prefer not to say

---

*Question type: **Single***
#Question display logic:
If [Q15h_5] - Yes is selected [if Q15h_5 == 1]

[Q15i_5] You said that you contacted your doctor (GP) again after noticing that you were still experiencing a **persistent change in bladder habits** after receiving your test results.

How long after you noticed that you were still experiencing the symptom did you contact your doctor (GP) again? If you are unsure, please give your best guess.

<1>	Within 1 week
<2>	Within 2 weeks
<3>	Within 3 weeks
<4>	Within 1 month
<5>	Within 6 weeks
<6>	Within 3 months
<7>	Within 6 months
<99>	Prefer not to say

#Module display logic:
If [Q8] - A persistent unexplained pain (persistent means doesn't go away), Yes is selected [if Q8_6 == 1]

Question type: ***Text***

You said that you have experienced **persistent unexplained pain** in the last 6 months. We would now like to ask you a few more questions about this.

Base: All experiencing each symptom
Question type: ***Single***
#SPD Category: health

[Q12_6] Approximately when did you first notice this symptom? Please give your best guess.
Please select one answer.

Response Option List: Q12_list

Base: All experiencing each symptom
Question type: ***Single***
#SPD Category: health

[Q13_6] You said that you have experienced **persistent unexplained pain** in the last 6 months. We would now like to ask you a few more questions about this.

How concerned have you been that this symptom might be serious?
Please select one answer.

Response Option List: Q13_list

Base: All experiencing each symptom
*Question type: **Multiple***
#row order: randomize
#SPD Category: health

[Q14_6] You said that you have experienced **persistent unexplained pain** in the last 6 months. We would now like to ask you a few more questions about this.

What do you think caused this symptom?
Please select all that apply.

Response Option List: Q14_list

Base: All experiencing each symptom
*Question type: **Single***
#SPD Category: health

[Q15_6] You said that you have experienced **persistent unexplained pain** in the last 6 months. We would now like to ask you a few more questions about this.

How long after you first noticed the symptom did you contact the GP (doctor's surgery) about it? If you are unsure, please give your best guess.
Please select one answer.

Response Option List: Q15_list

Base: All who made an appointment
*Question type: **Single***
#SPD Category: health
#Question display logic:
if Q15_6 in [3,4,5,6,7,8,9]

[Q15a_6] You said that you contacted your doctor after experiencing **persistent unexplained pain** in the last 6 months. How long after first contacting the GP (doctor's surgery) did the appointment take place?

This may have been an appointment that was face-to-face or remote, such as over the phone, by video call or online messaging. This also includes if a medical/health professional called you back after completing an online/e-consultation form.

If you are unsure, please give your best guess.

<1>	On the same day
<2>	On the next day
<3>	A few days later
<4>	A week later
<5>	Two weeks later
<6>	Three weeks later
<7>	Four or more weeks later
<8>	I have not had an appointment yet
<9>	Prefer not to say

Base: All who made an appointment
*Question type: **Single***
#SPD Category: health
#Question display logic:
if Q15_6 in [3,4,5,6,7,8,9] and Q15a_6 != 8

[Q15b_6] You said that you have discussed your **persistent unexplained pain** with your doctor (GP). Did you continue to experience the symptom after you first discussed it with your doctor (GP)?

<1>	Yes, I continued to experience the symptom and it was the same as before
<2>	Yes, I continued to experience the symptom, but it got better
<3>	Yes, I continued to experience the symptom and it got worse
<4>	No, the symptom went away by itself
<5>	No, the symptom went away after taking medication
<95>	Other (open [Q15b_6_open]) [open] Please specify
<99>	Prefer not to say

Base: all who continued to experience the symptom after discussing it with their doctor
*Question type: **Single***
#SPD Category: health
#Question display logic:
If [Q15b_6] - Yes, I continued to experience the symptom and it was the same as before or Yes, I continued to experience the symptom, but it got better or Yes, I continued to experience the symptom and it got worse or Other, is selected [if Q15b_6 in [1,2,3,95]]

[Q15c_6] You said you were still experiencing **persistent unexplained pain** after discussing it with your doctor (GP). Did you contact your doctor again after noticing that you were still experiencing the symptom?

<1>	Yes
<2>	No, but I plan to
<3>	No, and I don’t plan to
<99>	Not sure
<100>	Prefer not to say

Base: all who contacted their doctor again
*Question type: **Single***
#SPD Category: health
#Question display logic:
If [Q15c_6] - Yes is selected [if Q15c_6 == 1]

[Q15d_6] You said that you contacted your doctor (GP) again after noticing that you were still experiencing **persistent unexplained pain** in the last 6 months.

How long after you noticed that you were still experiencing the symptom did you contact your doctor (GP) again? If you are unsure, please give your best guess.

<1>	Within 1 week
<2>	Within 2 weeks
<3>	Within 3 weeks
<4>	Within 1 month
<5>	Within 6 weeks
<6>	Within 3 months
<7>	Within 6 months
<99>	Prefer not to say

Base: all who contacted their doctor again
*Question type: **Single***
#SPD Category: health

[Q15e_6] You said that you have experienced **persistent unexplained pain** in the last 6 months. Have you had any tests or investigations to find out what is causing this symptom?

<1>	Yes
<2>	No, but I am waiting for a test
<3>	No, and I am not waiting for a test
<98>	Not sure / don’t know
<99>	Prefer not to say

Base: all who had a test
*Question type: **Single***
#SPD Category: health
#Question display logic:
If [Q15e_6] - Yes is selected [if Q15e_6 == 1]

[Q15f_6] You said that you had a test or investigation for **persistent unexplained pain** in the last 6 months. What were the results of your tests?

<1>	The results suggested there was nothing to worry about
<2>	The results meant I needed more tests (to investigate further)
<3>	The results meant I needed to be monitored and retested again later
<4>	The results led to a diagnosis
<5>	I have not received my test results yet

<6>	I don't remember what the test results were
<95>	Other (open [Q15f_6_open]) [open] Please specify
<99>	Prefer not to say

Base: all who had a test
Question type: ***Single***
#SPD Category: health
#Question display logic:
If [Q15f_6] - The results suggested there was nothing to worry about is selected [if Q15f_6 == 1]

[Q15g_6] Did you continue to experience the symptom after your test result said there was nothing to worry about?
Please select one answer.

<1>	Yes, I continued to experience the symptom and it was the same as before
<2>	Yes, I continued to experience the symptom, but it got better
<3>	Yes, I continued to experience the symptom and it got worse
<4>	No, the symptom went away by itself
<5>	No, the symptom went away after taking medication
<95>	Other (open [Q15g_6_open]) [open] Please specify
<99>	Prefer not to say

Question type: ***Single***
#Question display logic:
If [Q15g_6] - Yes, I continued to experience the symptom and it was the same as before or Yes, I continued to experience the symptom, but it got better or Yes, I continued to experience the symptom and it got worse or Other, is selected [if Q15g_6 in [1,2,3,95]]

[Q15h_6] You said you were still experiencing **persistent unexplained pain** after receiving your test results. Did you contact your doctor again after noticing that you were still experiencing the symptom?

<1>	Yes
<2>	No, but I plan to
<3>	No, and I don't plan to
<99>	Not sure
<100>	Prefer not to say

Question type: ***Single***
#Question display logic:
If [Q15h_6] - Yes is selected [if Q15h_6 == 1]

[Q15i_6] You said that you contacted your doctor (GP) again after noticing that you were still experiencing **persistent unexplained pain** after receiving your test results.

How long after you noticed that you were still experiencing the symptom did you contact your doctor (GP) again? If you are unsure, please give your best guess.

<1>	Within 1 week
<2>	Within 2 weeks
<3>	Within 3 weeks
<4>	Within 1 month
<5>	Within 6 weeks
<6>	Within 3 months
<7>	Within 6 months
<99>	Prefer not to say

#Module display logic:
If [Q8] - A persistent difficulty swallowing (persistent means doesn’t go away), Yes is selected [if Q8_7 == 1]

Question type: ***Text***

You said that you have experienced **a persistent difficulty swallowing** in the last 6 months. We would now like to ask you a few more questions about this.

Base: All experiencing each symptom
Question type: ***Single***
#SPD Category: health

[Q12_7] Approximately when did you first notice this symptom? Please give your best guess.
Please select one answer.

Response Option List: Q12_list

Base: All experiencing each symptom
Question type: ***Single***
#SPD Category: health

[Q13_7] You said that you have experienced **a persistent difficulty swallowing** in the last 6 months. We would now like to ask you a few more questions about this.

How concerned have you been that this symptom might be serious?
Please select one answer.

Response Option List: Q13_list

Base: All experiencing each symptom
*Question type: **Multiple***
#row order: randomize
#SPD Category: health

[Q14_7] You said that you have experienced **a persistent difficulty swallowing** in the last 6 months. We would now like to ask you a few more questions about this.

What do you think caused this symptom?
Please select all that apply.

Response Option List: Q14_list

Base: All experiencing each symptom
*Question type: **Single***
#SPD Category: health

[Q15_7] You said that you have experienced **a persistent difficulty swallowing** in the last 6 months. We would now like to ask you a few more questions about this.

How long after you first noticed the symptom did you contact the GP (doctor's surgery) about it? If you are unsure, please give your best guess.
Please select one answer.

Response Option List: Q15_list

Base: All who made an appointment
*Question type: **Single***
#SPD Category: health
#Question display logic:
if Q15_7 in [3,4,5,6,7,8,9]

[Q15a_7] You said that you contacted your doctor after experiencing **a persistent difficulty swallowing** in the last 6 months. How long after first contacting the GP (doctor’s surgery) did the appointment take place?

This may have been an appointment that was face-to-face or remote, such as over the phone, by video call or online messaging. This also includes if a medical/health professional called you back after completing an online/e-consultation form.

If you are unsure, please give your best guess.

<1>	On the same day
<2>	On the next day
<3>	A few days later
<4>	A week later
<5>	Two weeks later
<6>	Three weeks later
<7>	Four or more weeks later
<8>	I have not had an appointment yet
<9>	Prefer not to say

Base: All who made an appointment
*Question type: **Single***
#SPD Category: health
#Question display logic:
if Q15_7 in [3,4,5,6,7,8,9] and Q15a_7 != 8

[Q15b_7] You said that you have discussed your **persistent difficulty swallowing** with your doctor (GP). Did you continue to experience the symptom after you first discussed it with your doctor (GP)?

<1>	Yes, I continued to experience the symptom and it was the same as before
<2>	Yes, I continued to experience the symptom, but it got better
<3>	Yes, I continued to experience the symptom and it got worse
<4>	No, the symptom went away by itself
<5>	No, the symptom went away after taking medication
<95>	Other (open [Q15b_7_open]) [open] Please specify
<99>	Prefer not to say

Base: all who continued to experience the symptom after discussing it with their doctor
*Question type: **Single***
#SPD Category: health
#Question display logic:
If [Q15b_7] - Yes, I continued to experience the symptom and it was the same as before or Yes, I continued to experience the symptom, but it got better or Yes, I continued to experience the symptom and it got worse or Other, is selected [if Q15b_7 in [1,2,3,95]]

[Q15c_7] You said you were still experiencing **a persistent difficulty swallowing** after discussing it with your doctor (GP). Did you contact your doctor again after noticing that you were still experiencing the symptom?

<1>	Yes
<2>	No, but I plan to
<3>	No, and I don't plan to
<99>	Not sure
<100>	Prefer not to say

Base: all who contacted their doctor again
*Question type: **Single***
#SPD Category: health
#Question display logic:
If [Q15c_7] - Yes is selected [if Q15c_7 == 1]

[Q15d_7] You said that you contacted your doctor (GP) again after noticing that you were still experiencing **a persistent difficulty swallowing** in the last 6 months.

How long after you noticed that you were still experiencing the symptom did you contact your doctor (GP) again? If you are unsure, please give your best guess.

<1>	Within 1 week
<2>	Within 2 weeks
<3>	Within 3 weeks
<4>	Within 1 month
<5>	Within 6 weeks
<6>	Within 3 months
<7>	Within 6 months
<99>	Prefer not to say

Base: all who contacted their doctor again
*Question type: **Single***
#SPD Category: health

[Q15e_7] You said that you have experienced **a persistent difficulty swallowing** in the last 6 months. Have you had any tests or investigations to find out what is causing this symptom?

<1>	Yes
<2>	No, but I am waiting for a test
<3>	No, and I am not waiting for a test
<98>	Not sure / don’t know
<99>	Prefer not to say

Base: all who had a test
*Question type: **Single***
#SPD Category: health
#Question display logic:
If [Q15e_7] - Yes is selected [if Q15e_7 == 1]

[Q15f_7] You said that you had a test or investigation for **a persistent difficulty swallowing** in the last 6 months. What were the results of your tests?

<1>	The results suggested there was nothing to worry about

<2>	The results meant I needed more tests (to investigate further)
<3>	The results meant I needed to be monitored and retested again later
<4>	The results led to a diagnosis
<5>	I have not received my test results yet
<6>	I don't remember what the test results were
<95>	Other (open [Q15f_7_open]) [open] Please specify
<99>	Prefer not to say

Base: all who had a test
Question type: ***Single***
#SPD Category: health
#Question display logic:
If [Q15f_7] - The results suggested there was nothing to worry about is selected [if Q15f_7 == 1]

[Q15g_7] Did you continue to experience the symptom after your test result said there was nothing to worry about?
Please select one answer.

<1>	Yes, I continued to experience the symptom and it was the same as before
<2>	Yes, I continued to experience the symptom, but it got better
<3>	Yes, I continued to experience the symptom and it got worse
<4>	No, the symptom went away by itself
<5>	No, the symptom went away after taking medication
<95>	Other (open [Q15g_7_open]) [open] Please specify
<99>	Prefer not to say

Question type: ***Single***
#Question display logic:
If [Q15g_7] - Yes, I continued to experience the symptom and it was the same as before or Yes, I continued to experience the symptom, but it got better or Yes, I continued to experience the symptom and it got worse or Other, is selected [if Q15g_7 in [1,2,3,95]]

[Q15h_7] You said you were still experiencing **a persistent difficulty swallowing** after receiving your test results. Did you contact your doctor again after noticing that you were still experiencing the symptom?

<1>	Yes
<2>	No, but I plan to
<3>	No, and I don't plan to
<99>	Not sure
<100>	Prefer not to say

---

*Question type: **Single***
#Question display logic:
If [Q15h_7] - Yes is selected [if Q15h_7 == 1]

[Q15i_7] You said that you contacted your doctor (GP) again after noticing that you were still experiencing **a persistent difficulty swallowing** after receiving your test results.

How long after you noticed that you were still experiencing the symptom did you contact your doctor (GP) again? If you are unsure, please give your best guess.

<1>	Within 1 week
<2>	Within 2 weeks
<3>	Within 3 weeks
<4>	Within 1 month
<5>	Within 6 weeks
<6>	Within 3 months
<7>	Within 6 months
<99>	Prefer not to say

#Module display logic:
If [Q8] - A persistent cough (persistent means doesn't go away), Yes is selected [if Q8_8 == 1]

---

*Question type: **Text***

You said that you have experienced **a persistent cough** in the last 6 months. We would now like to ask you a few more questions about this.

Base: All experiencing each symptom
*Question type: **Single***
#SPD Category: health

[Q12_8] Approximately when did you first notice this symptom? Please give your best guess.
Please select one answer.

Response Option List: Q12_list

Base: All experiencing each symptom
*Question type: **Single***
#SPD Category: health

[Q13_8] You said that you have experienced **a persistent cough** in the last 6 months. We would now like to ask you a few more questions about this.

How concerned have you been that this symptom might be serious?
Please select one answer.

Response Option List: Q13_list

Base: All experiencing each symptom
*Question type: **Multiple***
#row order: randomize
#SPD Category: health

[Q14_8] You said that you have experienced **a persistent cough** in the last 6 months. We would now like to ask you a few more questions about this.

What do you think caused this symptom?
Please select all that apply.

Response Option List: Q14_list

Base: All experiencing each symptom
*Question type: **Single***
#SPD Category: health

[Q15_8] You said that you have experienced **a persistent cough** in the last 6 months. We would now like to ask you a few more questions about this.

How long after you first noticed the symptom did you contact the GP (doctor's surgery) about it? If you are unsure, please give your best guess.
Please select one answer.

Response Option List: Q15_list

Base: All who made an appointment
*Question type: **Single***
#SPD Category: health
#Question display logic:
if Q15_8 in [3,4,5,6,7,8,9]

[Q15a_8] You said that you contacted your doctor after experiencing **a persistent cough** in the last 6 months. How long after first contacting the GP (doctor's surgery) did the appointment take place?

This may have been an appointment that was face-to-face or remote, such as over the phone, by video call or online messaging. This also includes if a medical/health professional called you back after completing an online/e-consultation form.

If you are unsure, please give your best guess.

<1>	On the same day
<2>	On the next day
<3>	A few days later
<4>	A week later
<5>	Two weeks later
<6>	Three weeks later
<7>	Four or more weeks later
<8>	I have not had an appointment yet
<9>	Prefer not to say

Base: All who made an appointment
*Question type: **Single***
#SPD Category: health
#Question display logic:
if Q15_8 in [3,4,5,6,7,8,9] and Q15a_8 != 8

[Q15b_8] You said that you have discussed **a persistent cough** with your doctor (GP). Did you continue to experience the symptom after you first discussed it with your doctor (GP)?

<1>	Yes, I continued to experience the symptom and it was the same as before
<2>	Yes, I continued to experience the symptom, but it got better
<3>	Yes, I continued to experience the symptom and it got worse
<4>	No, the symptom went away by itself
<5>	No, the symptom went away after taking medication
<95>	Other (open [Q15b_8_open]) [open] Please specify
<99>	Prefer not to say

Base: all who continued to experience the symptom after discussing it with their doctor
*Question type: **Single***
#SPD Category: health
#Question display logic:
If [Q15b_8] - Yes, I continued to experience the symptom and it was the same as before or Yes, I continued to experience the symptom, but it got better or Yes, I continued to experience the symptom and it got worse or Other, is selected [if Q15b_8 in [1,2,3,95]]

[Q15c_8] You said you were still experiencing **a persistent cough** after discussing it with your doctor (GP). Did you contact your doctor again after noticing that you were still experiencing the symptom?

<1> Yes
<2> No, but I plan to
<3> No, and I don’t plan to
<99> Not sure
<100> Prefer not to say

Base: all who contacted their doctor again
*Question type: **Single***
#SPD Category: health
#Question display logic:
If [Q15c_8] - Yes is selected [if Q15c_8 == 1]

[Q15d_8] You said that you contacted your doctor (GP) again after noticing that you were still experiencing **a persistent cough** in the last 6 months.

How long after you noticed that you were still experiencing the symptom did you contact your doctor (GP) again? If you are unsure, please give your best guess.

<1> Within 1 week
<2> Within 2 weeks
<3> Within 3 weeks
<4> Within 1 month
<5> Within 6 weeks
<6> Within 3 months
<7> Within 6 months
<99> Prefer not to say

Base: all who contacted their doctor again
*Question type: **Single***
#SPD Category: health

[Q15e_8] You said that you have experienced **a persistent cough** in the last 6 months. Have you had any tests or investigations to find out what is causing this symptom?

<1> Yes
<2> No, but I am waiting for a test
<3> No, and I am not waiting for a test
<98> Not sure / don’t know
<99> Prefer not to say

Base: all who had a test
*Question type: **Single***
#SPD Category: health
#Question display logic:
If [Q15e_8] - Yes is selected [if Q15e_8 == 1]

[Q15f_8] You said that you had a test or investigation for **a persistent cough** in the last 6 months. What were the results of your tests?

<1>	The results suggested there was nothing to worry about
<2>	The results meant I needed more tests (to investigate further)
<3>	The results meant I needed to be monitored and retested again later
<4>	The results led to a diagnosis
<5>	I have not received my test results yet
<6>	I don't remember what the test results were
<95>	Other (open [Q15f_8_open]) [open] Please specify
<99>	Prefer not to say

Base: all who had a test

Question type: ***Single***

#SPD Category: health

#Question display logic:

If [Q15f_8] - The results suggested there was nothing to worry about is selected [if Q15f_8 == 1]

[Q15g_8] Did you continue to experience the symptom after your test result said there was nothing to worry about?
Please select one answer.

<1>	Yes, I continued to experience the symptom and it was the same as before
<2>	Yes, I continued to experience the symptom, but it got better
<3>	Yes, I continued to experience the symptom and it got worse
<4>	No, the symptom went away by itself
<5>	No, the symptom went away after taking medication
<95>	Other (open [Q15g_8_open]) [open] Please specify
<99>	Prefer not to say

Question type: ***Single***

#Question display logic:

If [Q15g_8] - Yes, I continued to experience the symptom and it was the same as before or Yes, I continued to experience the symptom, but it got better or Yes, I continued to experience the symptom and it got worse or Other, is selected [if Q15g_8 in [1,2,3,95]]

[Q15h_8] You said you were still experiencing **a persistent cough** after receiving your test results. Did you contact your doctor again after noticing that you were still experiencing the symptom?

<1>	Yes
<2>	No, but I plan to
<3>	No, and I don't plan to
<99>	Not sure
<100>	Prefer not to say

Question type: **Single**
#Question display logic:
If [Q15h_8] - Yes is selected [if Q15h_8 == 1]

[Q15i_8] You said that you contacted your doctor (GP) again after noticing that you were still experiencing **a persistent cough** after receiving your test results.

How long after you noticed that you were still experiencing the symptom did you contact your doctor (GP) again? If you are unsure, please give your best guess.

<1>	Within 1 week
<2>	Within 2 weeks
<3>	Within 3 weeks
<4>	Within 1 month
<5>	Within 6 weeks
<6>	Within 3 months
<7>	Within 6 months
<99>	Prefer not to say

#Module display logic:
If [Q8] - Unexplained bleeding, Yes is selected [if Q8_9 == 1]

Question type: ***Text***

You said that you have experienced **unexplained bleeding** in the last 6 months. We would now like to ask you a few more questions about this.

Base: All experiencing each symptom
Question type: ***Single***
#SPD Category: health

[Q10] Where did the bleeding come from?
Please select one answer.

<1>	Blood in your poo or from your back passage
<2>	Blood in your wee
<3>	Blood from your vagina
<4 fixed>	Other (Please specify)
<5 fixed>	Prefer not to say

Base: All experiencing each symptom
*Question type: **Single***
#SPD Category: health

[Q12_9] You said that you have experienced **unexplained bleeding** in the last 6 months. We would now like to ask you a few more questions about this.

Approximately when did you first notice this symptom? Please give your best guess.
Please select one answer.

Response Option List: Q12_list

Base: All experiencing each symptom
*Question type: **Single***
#SPD Category: health

[Q13_9] You said that you have experienced **unexplained bleeding** in the last 6 months. We would now like to ask you a few more questions about this.

How concerned have you been that this symptom might be serious?
Please select one answer.

Response Option List: Q13_list

Base: All experiencing each symptom
*Question type: **Multiple***
#row order: randomize
#SPD Category: health

[Q14_9] You said that you have experienced **unexplained bleeding** in the last 6 months. We would now like to ask you a few more questions about this.

What do you think caused this symptom?
Please select all that apply.

Response Option List: Q14_list

Base: All experiencing each symptom
*Question type: **Single***
#SPD Category: health

[Q15_9] You said that you have experienced **unexplained bleeding** in the last 6 months. We would now like to ask you a few more questions about this.

How long after you first noticed the symptom did you contact the GP (doctor's surgery) about it? If you are unsure, please give your best guess.
Please select one answer.

Response Option List: Q15_list

Base: All who made an appointment
*Question type: **Single***
#SPD Category: health
#Question display logic:
if Q15_9 in [3,4,5,6,7,8,9]

[Q15a_9] You said that you contacted your doctor after experiencing **unexplained bleeding** in the last 6 months. How long after first contacting the GP (doctor's surgery) did the appointment take place?

This may have been an appointment that was face-to-face or remote, such as over the phone, by video call or online messaging. This also includes if a medical/health professional called you back after completing an online/e-consultation form.

If you are unsure, please give your best guess.

<1>	On the same day
<2>	On the next day
<3>	A few days later
<4>	A week later
<5>	Two weeks later
<6>	Three weeks later
<7>	Four or more weeks later
<8>	I have not had an appointment yet
<9>	Prefer not to say

Base: All who made an appointment
*Question type: **Single***
#SPD Category: health
#Question display logic:
if Q15_9 in [3,4,5,6,7,8,9] and Q15a_9 != 8

[Q15b_9] You said that you have discussed the **unexplained bleeding** with your doctor (GP). Did you continue to experience the symptom after you first discussed it with your doctor (GP)?

<1>	Yes, I continued to experience the symptom and it was the same as before
<2>	Yes, I continued to experience the symptom, but it got better

<3>	Yes, I continued to experience the symptom and it got worse
<4>	No, the symptom went away by itself
<5>	No, the symptom went away after taking medication
<95>	Other (open [Q15b_9_open]) [open] Please specify
<99>	Prefer not to say

Base: all who continued to experience the symptom after discussing it with their doctor
*Question type: **Single***
#SPD Category: health
#Question display logic:
If [Q15b_9] - Yes, I continued to experience the symptom and it was the same as before or Yes, I continued to experience the symptom, but it got better or Yes, I continued to experience the symptom and it got worse or Other, is selected [if Q15b_9 in [1,2,3,95]]

[Q15c_9] You said you were still experiencing **unexplained bleeding** after discussing it with your doctor (GP). Did you contact your doctor again after noticing that you were still experiencing the symptom?

<1>	Yes
<2>	No, but I plan to
<3>	No, and I don’t plan to
<99>	Not sure
<100>	Prefer not to say

Base: all who contacted their doctor again
*Question type: **Single***
#SPD Category: health
#Question display logic:
If [Q15c_9] - Yes is selected [if Q15c_9 == 1]

[Q15d_9] You said that you contacted your doctor (GP) again after noticing that you were still experiencing **unexplained bleeding** in the last 6 months.

How long after you noticed that you were still experiencing the symptom did you contact your doctor (GP) again? If you are unsure, please give your best guess.

<1>	Within 1 week
<2>	Within 2 weeks
<3>	Within 3 weeks
<4>	Within 1 month
<5>	Within 6 weeks
<6>	Within 3 months
<7>	Within 6 months
<99>	Prefer not to say

Base: all who contacted their doctor again
*Question type: **Single***
#SPD Category: health

[Q15e_9] You said that you have experienced **unexplained bleeding** in the last 6 months. Have you had any tests or investigations to find out what is causing this symptom?

<1>	Yes
<2>	No, but I am waiting for a test
<3>	No, and I am not waiting for a test
<98>	Not sure / don't know
<99>	Prefer not to say

Base: all who had a test
*Question type: **Single***
#SPD Category: health
#Question display logic:
If [Q15e_9] - Yes is selected [if Q15e_9 == 1]

[Q15f_9] You said that you had a test or investigation for **unexplained bleeding** in the last 6 months. What were the results of your tests?

<1>	The results suggested there was nothing to worry about
<2>	The results meant I needed more tests (to investigate further)
<3>	The results meant I needed to be monitored and retested again later
<4>	The results led to a diagnosis
<5>	I have not received my test results yet
<6>	I don't remember what the test results were
<95>	Other (open [Q15f_9_open]) [open] Please specify
<99>	Prefer not to say

Base: all who had a test
*Question type: **Single***
#SPD Category: health
#Question display logic:
If [Q15f_9] - The results suggested there was nothing to worry about is selected [if Q15f_9 == 1]

[Q15g_9] Did you continue to experience the symptom after your test result said there was nothing to worry about?
Please select one answer.

<1>	Yes, I continued to experience the symptom and it was the same as before
<2>	Yes, I continued to experience the symptom, but it got better
<3>	Yes, I continued to experience the symptom and it got worse
<4>	No, the symptom went away by itself
<5>	No, the symptom went away after taking medication
<95>	Other (open [Q15g_9_open]) [open] Please specify
<99>	Prefer not to say

Question type: ***Single***
#Question display logic:
If [Q15g_9] - Yes, I continued to experience the symptom and it was the same as before or Yes, I continued to experience the symptom, but it got better or Yes, I continued to experience the symptom and it got worse or Other, is selected [if Q15g_9 in [1,2,3,95]]

[Q15h_9] You said you were still experiencing **unexplained bleeding** after receiving your test results. Did you contact your doctor again after noticing that you were still experiencing the symptom?

<1>	Yes
<2>	No, but I plan to
<3>	No, and I don’t plan to
<99>	Not sure
<100>	Prefer not to say

Question type: ***Single***
#Question display logic:
If [Q15h_9] - Yes is selected [if Q15h_9 == 1]

[Q15i_9] You said that you contacted your doctor (GP) again after noticing that you were still experiencing **unexplained bleeding** after receiving your test results.

How long after you noticed that you were still experiencing the symptom did you contact your doctor (GP) again? If you are unsure, please give your best guess.

<1>	Within 1 week
<2>	Within 2 weeks
<3>	Within 3 weeks
<4>	Within 1 month
<5>	Within 6 weeks
<6>	Within 3 months
<7>	Within 6 months
<99>	Prefer not to say

#Module display logic:
If [Q8] - A sore that does not heal, Yes is selected [if Q8_10 == 1]

Question type: ***Text***

You said that you have experienced **a sore that does not heal** in the last 6 months. We would now like to ask you a few more questions about this.

Base: All experiencing each symptom
*Question type: **Single***
#SPD Category: health

[Q11] Where on your body was the sore?
Please select one answer.

<1>	Skin
<2>	In/around your mouth
<3>	Genitals
<4>	Other
<5>	Prefer not to say

Base: All experiencing each symptom
*Question type: **Single***
#SPD Category: health

[Q12_10] You said that you have experienced **a sore that does not heal** in the last 6 months. We would now like to ask you a few more questions about this.

Approximately when did you first notice this symptom? Please give your best guess.
Please select one answer.

Response Option List: Q12_list

Base: All experiencing each symptom
*Question type: **Single***
#SPD Category: health

[Q13_10] You said that you have experienced **a sore that does not heal** in the last 6 months. We would now like to ask you a few more questions about this.

How concerned have you been that this symptom might be serious?
Please select one answer.

Response Option List: Q13_list

Base: All experiencing each symptom
*Question type: **Multiple***
#row order: randomize
#SPD Category: health

[Q14_10] You said that you have experienced **a sore that does not heal** in the last 6 months. We would now like to ask you a few more questions about this.

What do you think caused this symptom?
Please select all that apply.

Response Option List: Q14_list

Base: All experiencing each symptom
*Question type: **Single***
#SPD Category: health

[Q15_10] You said that you have experienced **a sore that does not heal** in the last 6 months. We would now like to ask you a few more questions about this.

How long after you first noticed the symptom did you contact the GP (doctor's surgery) about it? If you are unsure, please give your best guess.
Please select one answer.

Response Option List: Q15_list

Base: All who made an appointment
*Question type: **Single***
#SPD Category: health
#Question display logic:
if Q15_10 in [3,4,5,6,7,8,9]

[Q15a_10] You said that you contacted your doctor after experiencing **a sore that does not heal** in the last 6 months. How long after first contacting the GP (doctor's surgery) did the appointment take place?

This may have been an appointment that was face-to-face or remote, such as over the phone, by video call or online messaging. This also includes if a medical/health professional called you back after completing an online/e-consultation form.

If you are unsure, please give your best guess.

<1>	On the same day

<2>	On the next day
<3>	A few days later
<4>	A week later
<5>	Two weeks later
<6>	Three weeks later
<7>	Four or more weeks later
<8>	I have not had an appointment yet
<9>	Prefer not to say

Base: All who made an appointment
*Question type: **Single***
#SPD Category: health
#Question display logic:
if Q15_10 in [3,4,5,6,7,8,9] and Q15a_10 != 8

[Q15b_10] You said that you have discussed **a sore that does not heal** with your doctor (GP). Did you continue to experience the symptom after you first discussed it with your doctor (GP)?

<1>	Yes, I continued to experience the symptom and it was the same as before
<2>	Yes, I continued to experience the symptom, but it got better
<3>	Yes, I continued to experience the symptom and it got worse
<4>	No, the symptom went away by itself
<5>	No, the symptom went away after taking medication
<95>	Other (open [Q15b_10_open]) [open] Please specify
<99>	Prefer not to say

Base: all who continued to experience the symptom after discussing it with their doctor
*Question type: **Single***
#SPD Category: health
#Question display logic:
If [Q15b_10] - Yes, I continued to experience the symptom and it was the same as before or Yes, I continued to experience the symptom, but it got better or Yes, I continued to experience the symptom and it got worse or Other, is selected [if Q15b_10 in [1,2,3,95]]

[Q15c_10] You said you were still experiencing **a sore that does not heal** after discussing it with your doctor (GP). Did you contact your doctor again after noticing that you were still experiencing the symptom?

<1>	Yes
<2>	No, but I plan to
<3>	No, and I don’t plan to
<99>	Not sure
<100>	Prefer not to say

Base: all who contacted their doctor again
*Question type: **Single***
#SPD Category: health
#Question display logic:
If [Q15c_10] - Yes is selected [if Q15c_10 == 1]

[Q15d_10] You said that you contacted your doctor (GP) again after noticing that you were still experiencing **a sore that does not heal** in the last 6 months.

How long after you noticed that you were still experiencing the symptom did you contact your doctor (GP) again? If you are unsure, please give your best guess.

<1>	Within 1 week
<2>	Within 2 weeks
<3>	Within 3 weeks
<4>	Within 1 month
<5>	Within 6 weeks
<6>	Within 3 months
<7>	Within 6 months
<99>	Prefer not to say

Base: all who contacted their doctor again
*Question type: **Single***
#SPD Category: health

[Q15e_10] You said that you have experienced **a sore that does not heal** in the last 6 months. Have you had any tests or investigations to find out what is causing this symptom?

<1>	Yes
<2>	No, but I am waiting for a test
<3>	No, and I am not waiting for a test
<98>	Not sure / don’t know
<99>	Prefer not to say

Base: all who had a test
*Question type: **Single***
#SPD Category: health
#Question display logic:
If [Q15e_10] - Yes is selected [if Q15e_10 == 1]

[Q15f_10] You said that you had a test or investigation for **a sore that does not heal** in the last 6 months. What were the results of your tests?

<1>	The results suggested there was nothing to worry about
<2>	The results meant I needed more tests (to investigate further)
<3>	The results meant I needed to be monitored and retested again later
<4>	The results led to a diagnosis
<5>	I have not received my test results yet
<6>	I don’t remember what the test results were

<95>	Other (open [Q15f_10_open]) [open] Please specify
<99>	Prefer not to say

Base: all who had a test
Question type: ***Single***
#SPD Category: health
#Question display logic:
If [Q15f_10] - The results suggested there was nothing to worry about is selected [if Q15f_10 == 1]

[Q15g_10] Did you continue to experience **a sore that does not heal** after your test result said there was nothing to worry about?
Please select one answer.

<1>	Yes, I continued to experience the symptom and it was the same as before
<2>	Yes, I continued to experience the symptom, but it got better
<3>	Yes, I continued to experience the symptom and it got worse
<4>	No, the symptom went away by itself
<5>	No, the symptom went away after taking medication
<95>	Other (open [Q15g_10_open]) [open] Please specify
<99>	Prefer not to say

Question type: ***Single***
#Question display logic:
If [Q15g_10] - Yes, I continued to experience the symptom and it was the same as before or Yes, I continued to experience the symptom, but it got better or Yes, I continued to experience the symptom and it got worse or Other, is selected [if Q15g_10 in [1,2,3,95]]

[Q15h_10] You said you were still experiencing **a sore that does not heal** after receiving your test results. Did you contact your doctor again after noticing that you were still experiencing the symptom?

<1>	Yes
<2>	No, but I plan to
<3>	No, and I don’t plan to
<99>	Not sure
<100>	Prefer not to say

Question type: ***Single***
#Question display logic:
If [Q15h_10] - Yes is selected [if Q15h_10 == 1]

[Q15i_10] You said that you contacted your doctor (GP) again after noticing that you were still experiencing **a sore that does not heal** after receiving your test results.

How long after you noticed that you were still experiencing the symptom did you contact your doctor (GP) again? If you are unsure, please give your best guess.

<1>	Within 1 week
<2>	Within 2 weeks
<3>	Within 3 weeks
<4>	Within 1 month
<5>	Within 6 weeks
<6>	Within 3 months
<7>	Within 6 months
<99>	Prefer not to say

#Module display logic:
If [Q8] - Persistent hoarseness (persistent means doesn't go away), Yes is selected [if Q8_11 == 1]

Question type: ***Text***

You said that you have experienced **persistent hoarseness** in the last 6 months. We would now like to ask you a few more questions about this.

Base: All experiencing each symptom
Question type: ***Single***
#SPD Category: health

[Q12_11] Approximately when did you first notice this symptom? Please give your best guess.
Please select one answer.

Response Option List: Q12_list

Base: All experiencing each symptom
Question type: ***Single***
#SPD Category: health

[Q13_11] You said that you have experienced **persistent hoarseness** in the last 6 months. We would now like to ask you a few more questions about this.

How concerned have you been that this symptom might be serious?
Please select one answer.

Response Option List: Q13_list

Base: All experiencing each symptom
*Question type: **Multiple***
#row order: randomize
#SPD Category: health

[Q14_11] You said that you have experienced **persistent hoarseness** in the last 6 months. We would now like to ask you a few more questions about this.

What do you think caused this symptom?
Please select all that apply.

Response Option List: Q14_list

Base: All experiencing each symptom
*Question type: **Single***
#SPD Category: health

[Q15_11] You said that you have experienced **persistent hoarseness** in the last 6 months. We would now like to ask you a few more questions about this.

How long after you first noticed the symptom did you contact the GP (doctor's surgery) about it? If you are unsure, please give your best guess.
Please select one answer.

Response Option List: Q15_list

Base: All who made an appointment
*Question type: **Single***
#SPD Category: health
#Question display logic:
if Q15_11 in [3,4,5,6,7,8,9]

[Q15a_11] You said that you contacted your doctor after experiencing **persistent hoarseness** in the last 6 months. How long after first contacting the GP (doctor’s surgery) did the appointment take place?

This may have been an appointment that was face-to-face or remote, such as over the phone, by video call or online messaging. This also includes if a medical/health professional called you back after completing an online/e-consultation form.

If you are unsure, please give your best guess.

<1>	On the same day
<2>	On the next day
<3>	A few days later
<4>	A week later
<5>	Two weeks later
<6>	Three weeks later
<7>	Four or more weeks later
<8>	I have not had an appointment yet
<9>	Prefer not to say

Base: All who made an appointment
*Question type: **Single***
#SPD Category: health
#Question display logic:
if Q15_11 in [3,4,5,6,7,8,9] and Q15a_11 != 8

[Q15b_11] You said that you have discussed your **persistent hoarseness** with your doctor (GP). Did you continue to experience the symptom after you first discussed it with your doctor (GP)?

<1>	Yes, I continued to experience the symptom and it was the same as before
<2>	Yes, I continued to experience the symptom, but it got better
<3>	Yes, I continued to experience the symptom and it got worse
<4>	No, the symptom went away by itself
<5>	No, the symptom went away after taking medication
<95>	Other (open [Q15b_11_open]) [open] Please specify
<99>	Prefer not to say

Base: all who continued to experience the symptom after discussing it with their doctor
*Question type: **Single***
#SPD Category: health
#Question display logic:
If [Q15b_11] - Yes, I continued to experience the symptom and it was the same as before or Yes, I continued to experience the symptom, but it got better or Yes, I continued to experience the symptom and it got worse or Other, is selected [if Q15b_11 in [1,2,3,95]]

[Q15c_11] You said you were still experiencing **persistent hoarseness** after discussing it with your doctor (GP). Did you contact your doctor again after noticing that you were still experiencing the symptom?

<1>	Yes
<2>	No, but I plan to

<3>	No, and I don't plan to
<99>	Not sure
<100>	Prefer not to say

Base: all who contacted their doctor again
*Question type: **Single***
#SPD Category: health
#Question display logic:
If [Q15c_11] - Yes is selected [if Q15c_11 == 1]

[Q15d_11] You said that you contacted your doctor (GP) again after noticing that you were still experiencing **persistent hoarseness** in the last 6 months.

How long after you noticed that you were still experiencing the symptom did you contact your doctor (GP) again? If you are unsure, please give your best guess.

<1>	Within 1 week
<2>	Within 2 weeks
<3>	Within 3 weeks
<4>	Within 1 month
<5>	Within 6 weeks
<6>	Within 3 months
<7>	Within 6 months
<99>	Prefer not to say

Base: all who contacted their doctor again
*Question type: **Single***
#SPD Category: health

[Q15e_11] You said that you have experienced **persistent hoarseness** in the last 6 months. Have you had any tests or investigations to find out what is causing this symptom?

<1>	Yes
<2>	No, but I am waiting for a test
<3>	No, and I am not waiting for a test
<98>	Not sure / don't know
<99>	Prefer not to say

Base: all who had a test
*Question type: **Single***
#SPD Category: health
#Question display logic:
If [Q15e_11] - Yes is selected [if Q15e_11 == 1]

[Q15f_11] You said that you had a test or investigation for **persistent hoarseness** in the last 6 months. What were the results of your tests?

<1>	The results suggested there was nothing to worry about
<2>	The results meant I needed more tests (to investigate further)
<3>	The results meant I needed to be monitored and retested again later
<4>	The results led to a diagnosis
<5>	I have not received my test results yet
<6>	I don't remember what the test results were
<95>	Other (open [Q15f_11_open]) [open] Please specify
<99>	Prefer not to say

Base: all who had a test
*Question type: **Single***
#SPD Category: health
#Question display logic:
If [Q15f_11] - The results suggested there was nothing to worry about is selected [if Q15f_11 == 1]

[Q15g_11] Did you continue to experience the symptom after your test result said there was nothing to worry about?
Please select one answer.

<1>	Yes, I continued to experience the symptom and it was the same as before
<2>	Yes, I continued to experience the symptom, but it got better
<3>	Yes, I continued to experience the symptom and it got worse
<4>	No, the symptom went away by itself
<5>	No, the symptom went away after taking medication
<95>	Other (open [Q15g_11_open]) [open] Please specify
<99>	Prefer not to say

---

*Question type: **Single***
#Question display logic:
If [Q15g_11] - Yes, I continued to experience the symptom and it was the same as before or Yes, I continued to experience the symptom, but it got better or Yes, I continued to experience the symptom and it got worse or Other, is selected [if Q15g_11 in [1,2,3,95]]

[Q15h_11] You said you were still experiencing **persistent hoarseness** after receiving your test results. Did you contact your doctor again after noticing that you were still experiencing the symptom?

<1>	Yes
<2>	No, but I plan to
<3>	No, and I don't plan to
<99>	Not sure
<100>	Prefer not to say

Question type: **Single**
#Question display logic:
If [Q15h_11] - Yes is selected [if Q15h_11 == 1]

[Q15i_11] You said that you contacted your doctor (GP) again after noticing that you were still experiencing **persistent hoarseness** after receiving your test results.

How long after you noticed that you were still experiencing the symptom did you contact your doctor (GP) again? If you are unsure, please give your best guess.

<1>	Within 1 week
<2>	Within 2 weeks
<3>	Within 3 weeks
<4>	Within 1 month
<5>	Within 6 weeks
<6>	Within 3 months
<7>	Within 6 months
<99>	Prefer not to say

#Module display logic:
If [Q8] - Coughing up blood, Yes is selected [if Q8_12 == 1]

Question type: **Text**

You said that you have experienced **coughing up blood** in the last 6 months. We would now like to ask you a few more questions about this.

Base: All experiencing each symptom
Question type: **Single**
#SPD Category: health

[Q12_12] Approximately when did you first notice this symptom? Please give your best guess.
Please select one answer.

Response Option List: Q12_list

Base: All experiencing each symptom
Question type: **Single**
#SPD Category: health

[Q13_12] You said that you have experienced **coughing up blood** in the last 6 months. We would now like to ask you a few more questions about this.

How concerned have you been that this symptom might be serious?
Please select one answer.

Response Option List: Q13_list

Base: All experiencing each symptom
Question type: ***Multiple***
#row order: randomize
#SPD Category: health

[Q14_12] You said that you have experienced **coughing up blood** in the last 6 months. We would now like to ask you a few more questions about this.

What do you think caused this symptom?
Please select all that apply.

Response Option List: Q14_list

Base: All experiencing each symptom
Question type: ***Single***
#SPD Category: health

[Q15_12] You said that you have experienced **coughing up blood** in the last 6 months. We would now like to ask you a few more questions about this.

How long after you first noticed the symptom did you contact the GP (doctor's surgery) about it? If you are unsure, please give your best guess.
Please select one answer.

Response Option List: Q15_list

Base: All who made an appointment
Question type: ***Single***
#SPD Category: health
#Question display logic:
if Q15_12 in [3,4,5,6,7,8,9]

[Q15a_12] You said that you contacted your doctor after experiencing **coughing up blood** in the last 6 months. How long after first contacting the GP (doctor's surgery) did the appointment take place?

This may have been an appointment that was face-to-face or remote, such as over the phone, by video call or online messaging. This also includes if a medical/health professional called you back after completing an online/e-consultation form.

If you are unsure, please give your best guess.

<1>	On the same day
<2>	On the next day
<3>	A few days later
<4>	A week later
<5>	Two weeks later
<6>	Three weeks later
<7>	Four or more weeks later
<8>	I have not had an appointment yet
<9>	Prefer not to say

Base: All who made an appointment
*Question type: **Single***
#SPD Category: health
#Question display logic:
if Q15_12 in [3,4,5,6,7,8,9] and Q15a_12 != 8

[Q15b_12] You said that you have discussed **coughing up blood** with your doctor (GP). Did you continue to experience the symptom after you first discussed it with your doctor (GP)?

<1>	Yes, I continued to experience the symptom and it was the same as before
<2>	Yes, I continued to experience the symptom, but it got better
<3>	Yes, I continued to experience the symptom and it got worse
<4>	No, the symptom went away by itself
<5>	No, the symptom went away after taking medication
<95>	Other (open [Q15b_12_open]) [open] Please specify
<99>	Prefer not to say

Base: all who continued to experience the symptom after discussing it with their doctor
*Question type: **Single***
#SPD Category: health
#Question display logic:
If [Q15b_12] - Yes, I continued to experience the symptom and it was the same as before or Yes, I continued to experience the symptom, but it got better or Yes, I continued to experience the symptom and it got worse or Other, is selected [if Q15b_12 in [1,2,3,95]]

[Q15c_12] You said you were still **coughing up blood** after discussing it with your doctor (GP). Did you contact your doctor again after noticing that you were still experiencing the symptom?

<1>	Yes
<2>	No, but I plan to
<3>	No, and I don’t plan to
<99>	Not sure
<100>	Prefer not to say

Base: all who contacted their doctor again
*Question type: **Single***
#SPD Category: health
#Question display logic:
If [Q15c_12] - Yes is selected [if Q15c_12 == 1]

[Q15d_12] You said that you contacted your doctor (GP) again after noticing that you were still **coughing up blood** in the last 6 months.

How long after you noticed that you were still experiencing the symptom did you contact your doctor (GP) again? If you are unsure, please give your best guess.

<1>	Within 1 week
<2>	Within 2 weeks
<3>	Within 3 weeks
<4>	Within 1 month
<5>	Within 6 weeks
<6>	Within 3 months
<7>	Within 6 months
<99>	Prefer not to say

Base: all who contacted their doctor again
*Question type: **Single***
#SPD Category: health

[Q15e_12] You said that you have experienced **coughing up blood** in the last 6 months. Have you had any tests or investigations to find out what is causing this symptom?

<1>	Yes
<2>	No, but I am waiting for a test
<3>	No, and I am not waiting for a test
<98>	Not sure / don’t know
<99>	Prefer not to say

Base: all who had a test
*Question type: **Single***
#SPD Category: health
#Question display logic:
If [Q15e_12] - Yes is selected [if Q15e_12 == 1]

[Q15f_12] You said that you had a test or investigation for **coughing up blood** in the last 6 months. What were the results of your tests?

<1>	The results suggested there was nothing to worry about
<2>	The results meant I needed more tests (to investigate further)
<3>	The results meant I needed to be monitored and retested again later
<4>	The results led to a diagnosis
<5>	I have not received my test results yet
<6>	I don't remember what the test results were
<95>	Other (open [Q15f_12_open]) [open] Please specify
<99>	Prefer not to say

Base: all who had a test
Question type: ***Single***
#SPD Category: health
#Question display logic:
If [Q15f_12] - The results suggested there was nothing to worry about is selected [if Q15f_12 == 1]

[Q15g_12] Did you continue to experience the symptom after your test result said there was nothing to worry about?
Please select one answer.

<1>	Yes, I continued to experience the symptom and it was the same as before
<2>	Yes, I continued to experience the symptom, but it got better
<3>	Yes, I continued to experience the symptom and it got worse
<4>	No, the symptom went away by itself
<5>	No, the symptom went away after taking medication
<95>	Other (open [Q15g_12_open]) [open] Please specify
<99>	Prefer not to say

Question type: ***Single***
#Question display logic:
If [Q15g_12] - Yes, I continued to experience the symptom and it was the same as before or Yes, I continued to experience the symptom, but it got better or Yes, I continued to experience the symptom and it got worse or Other, is selected [if Q15g_12 in [1,2,3,95]]

[Q15h_12] You said you were still experiencing **coughing up blood** after receiving your test results. Did you contact your doctor again after noticing that you were still experiencing the symptom?

<1>	Yes
<2>	No, but I plan to
<3>	No, and I don't plan to
<99>	Not sure
<100>	Prefer not to say

Question type: ***Single***
#Question display logic:
If [Q15h_12] - Yes is selected [if Q15h_12 == 1]

[Q15i_12] You said that you contacted your doctor (GP) again after noticing that you were still experiencing **coughing up blood** after receiving your test results.

How long after you noticed that you were still experiencing the symptom did you contact your doctor (GP) again? If you are unsure, please give your best guess.

<1>	Within 1 week
<2>	Within 2 weeks
<3>	Within 3 weeks
<4>	Within 1 month
<5>	Within 6 weeks
<6>	Within 3 months
<7>	Within 6 months
<99>	Prefer not to say

#Module display logic:
If [Q8] - Feeling tired all the time, Yes is selected [if Q8_13 == 1]

Question type: ***Text***

You said that you have experienced **feeling tired all the time** in the last 6 months. We would now like to ask you a few more questions about this.

Base: All experiencing each symptom
Question type: ***Single***
#SPD Category: health

[Q12_13] Approximately when did you first notice this symptom? Please give your best guess.
Please select one answer.

Response Option List: Q12_list

Base: All experiencing each symptom
*Question type: **Single***
#SPD Category: health

[Q13_13] You said that you have experienced **feeling tired all the time** in the last 6 months. We would now like to ask you a few more questions about this.

How concerned have you been that this symptom might be serious?
Please select one answer.

Response Option List: Q13_list

Base: All experiencing each symptom
*Question type: **Multiple***
#row order: randomize
#SPD Category: health

[Q14_13] You said that you have experienced **feeling tired all the time** in the last 6 months. We would now like to ask you a few more questions about this.

What do you think caused this symptom?
Please select all that apply.

Response Option List: Q14_list

Base: All experiencing each symptom
*Question type: **Single***
#SPD Category: health

[Q15_13] You said that you have experienced **feeling tired all the time** in the last 6 months. We would now like to ask you a few more questions about this.

How long after you first noticed the symptom did you contact the GP (doctor's surgery) about it? If you are unsure, please give your best guess.
Please select one answer.

Response Option List: Q15_list

Base: All who made an appointment
*Question type: **Single***
#SPD Category: health

#Question display logic:
if Q15_13 in [3,4,5,6,7,8,9]

[Q15a_13] You said that you contacted your doctor after experiencing **feeling tired all the time** in the last 6 months. How long after first contacting the GP (doctor's surgery) did the appointment take place?

This may have been an appointment that was face-to-face or remote, such as over the phone, by video call or online messaging. This also includes if a medical/health professional called you back after completing an online/e-consultation form.

If you are unsure, please give your best guess.

<1>	On the same day
<2>	On the next day
<3>	A few days later
<4>	A week later
<5>	Two weeks later
<6>	Three weeks later
<7>	Four or more weeks later
<8>	I have not had an appointment yet
<9>	Prefer not to say

Base: All who made an appointment
Question type: ***Single***
#SPD Category: health
#Question display logic:
if Q15_13 in [3,4,5,6,7,8,9] and Q15a_13 != 8

[Q15b_13] You said that you have discussed your experience of **feeling tired all the time** with your doctor (GP). Did you continue to experience the symptom after you first discussed it with your doctor (GP)?

<1>	Yes, I continued to experience the symptom and it was the same as before
<2>	Yes, I continued to experience the symptom, but it got better
<3>	Yes, I continued to experience the symptom and it got worse
<4>	No, the symptom went away by itself
<5>	No, the symptom went away after taking medication
<95>	Other (open [Q15b_13_open]) [open] Please specify
<99>	Prefer not to say

Base: all who continued to experience the symptom after discussing it with their doctor
Question type: ***Single***
#SPD Category: health
#Question display logic:
If [Q15b_13] - Yes, I continued to experience the symptom and it was the same as before or Yes, I continued to experience the symptom, but it got better or Yes, I continued to experience the symptom and it got worse or Other, is selected [if Q15b_13 in [1,2,3,95]]

[Q15c_13] You said you were still experiencing **feeling tired all the time** after discussing it with your doctor (GP). Did you contact your doctor again after noticing that you were still experiencing the symptom?

<1>	Yes
<2>	No, but I plan to
<3>	No, and I don’t plan to
<99>	Not sure
<100>	Prefer not to say

Base: all who contacted their doctor again
*Question type: **Single***
#SPD Category: health
#Question display logic:
If [Q15c_13] - Yes is selected [if Q15c_13 == 1]

[Q15d_13] You said that you contacted your doctor (GP) again after noticing that you were still experiencing **feeling tired all the time** in the last 6 months.

How long after you noticed that you were still experiencing the symptom did you contact your doctor (GP) again? If you are unsure, please give your best guess.

<1>	Within 1 week
<2>	Within 2 weeks
<3>	Within 3 weeks
<4>	Within 1 month
<5>	Within 6 weeks
<6>	Within 3 months
<7>	Within 6 months
<99>	Prefer not to say

Base: all who contacted their doctor again
*Question type: **Single***
#SPD Category: health

[Q15e_13] You said that you have experienced **feeling tired all the time** in the last 6 months. Have you had any tests or investigations to find out what is causing this symptom?

<1>	Yes
<2>	No, but I am waiting for a test
<3>	No, and I am not waiting for a test
<98>	Not sure / don’t know
<99>	Prefer not to say

Base: all who had a test
*Question type: **Single***

#SPD Category: health
#Question display logic:
If [Q15e_13] - Yes is selected [if Q15e_13 == 1]

[Q15f_13] You said that you had a test or investigation for **feeling tired all the time** in the last 6 months. What were the results of your tests?

<1>	The results suggested there was nothing to worry about
<2>	The results meant I needed more tests (to investigate further)
<3>	The results meant I needed to be monitored and retested again later
<4>	The results led to a diagnosis
<5>	I have not received my test results yet
<6>	I don't remember what the test results were
<95>	Other (open [Q15f_13_open]) [open] Please specify
<99>	Prefer not to say

Base: all who had a test
Question type: ***Single***
#SPD Category: health
#Question display logic:
If [Q15f_13] - The results suggested there was nothing to worry about is selected [if Q15f_13 == 1]

[Q15g_13] Did you continue to experience the symptom after your test result said there was nothing to worry about?
Please select one answer.

<1>	Yes, I continued to experience the symptom and it was the same as before
<2>	Yes, I continued to experience the symptom, but it got better
<3>	Yes, I continued to experience the symptom and it got worse
<4>	No, the symptom went away by itself
<5>	No, the symptom went away after taking medication
<95>	Other (open [Q15g_13_open]) [open] Please specify
<99>	Prefer not to say

Question type: ***Single***
#Question display logic:
If [Q15g_13] - Yes, I continued to experience the symptom and it was the same as before or Yes, I continued to experience the symptom, but it got better or Yes, I continued to experience the symptom and it got worse or Other, is selected [if Q15g_13 in [1,2,3,95]]

[Q15h_13] You said you were still experiencing **feeling tired all the time** after receiving your test results. Did you contact your doctor again after noticing that you were still experiencing the symptom?

<1>	Yes
<2>	No, but I plan to
<3>	No, and I don't plan to

<99> Not sure
<100> Prefer not to say

---

*Question type: **Single***
#Question display logic:
If [Q15h_13] - Yes is selected [if Q15h_13 == 1]

[Q15i_13] You said that you contacted your doctor (GP) again after noticing that you were still experiencing **feeling tired all the time** after receiving your test results.

How long after you noticed that you were still experiencing the symptom did you contact your doctor (GP) again? If you are unsure, please give your best guess.

<1>	Within 1 week
<2>	Within 2 weeks
<3>	Within 3 weeks
<4>	Within 1 month
<5>	Within 6 weeks
<6>	Within 3 months
<7>	Within 6 months
<99>	Prefer not to say

#Module display logic:
If [Q8] - A change in an existing cough, Yes is selected [if Q8_14 == 1]

---

*Question type: **Text***

You said that you have experienced **a change in an existing cough** in the last 6 months. We would now like to ask you a few more questions about this.

Base: All experiencing each symptom
*Question type: **Single***
#SPD Category: health

[Q12_14] Approximately when did you first notice this symptom? Please give your best guess.
Please select one answer.

Response Option List: Q12_list

Base: All experiencing each symptom
*Question type: **Single***
#SPD Category: health

[Q13_14] You said that you have experienced **a change in an existing cough** in the last 6 months. We would now like to ask you a few more questions about this.

How concerned have you been that this symptom might be serious?
Please select one answer.

Response Option List: Q13_list

Base: All experiencing each symptom
*Question type: **Multiple***
#row order: randomize
#SPD Category: health

[Q14_14] You said that you have experienced **a change in an existing cough** in the last 6 months. We would now like to ask you a few more questions about this.

What do you think caused this symptom?
Please select all that apply.

Response Option List: Q14_list

Base: All experiencing each symptom
*Question type: **Single***
#SPD Category: health

[Q15_14] You said that you have experienced **a change in an existing cough** in the last 6 months. We would now like to ask you a few more questions about this.

How long after you first noticed the symptom did you contact the GP (doctor's surgery) about it? If you are unsure, please give your best guess.
Please select one answer.

Response Option List: Q15_list

Base: All who made an appointment
*Question type: **Single***
#SPD Category: health
#Question display logic:
if Q15_14 in [3,4,5,6,7,8,9]

[Q15a_14] You said that you contacted your doctor after experiencing **a change in an existing cough** in the last 6 months. How long after first contacting the GP (doctor's surgery) did the appointment take place?

This may have been an appointment that was face-to-face or remote, such as over the phone, by video call or online messaging. This also includes if a medical/health professional called you back after completing an online/e-consultation form.

If you are unsure, please give your best guess.

<1>	On the same day
<2>	On the next day
<3>	A few days later
<4>	A week later
<5>	Two weeks later
<6>	Three weeks later
<7>	Four or more weeks later
<8>	I have not had an appointment yet
<9>	Prefer not to say

Base: All who made an appointment
*Question type: **Single***
#SPD Category: health
#Question display logic:
if Q15_14 in [3,4,5,6,7,8,9] and Q15a_14 != 8

[Q15b_14] You said that you have discussed **a change in an existing cough** with your doctor (GP). Did you continue to experience the symptom after you first discussed it with your doctor (GP)?

<1>	Yes, I continued to experience the symptom and it was the same as before
<2>	Yes, I continued to experience the symptom, but it got better
<3>	Yes, I continued to experience the symptom and it got worse
<4>	No, the symptom went away by itself
<5>	No, the symptom went away after taking medication
<95>	Other (open [Q15b_14_open]) [open] Please specify
<99>	Prefer not to say

Base: all who continued to experience the symptom after discussing it with their doctor
*Question type: **Single***
#SPD Category: health
#Question display logic:
If [Q15b_14] - Yes, I continued to experience the symptom and it was the same as

before or Yes, I continued to experience the symptom, but it got better or Yes, I continued to experience the symptom and it got worse or Other, is selected [if Q15b_14 in [1,2,3,95]]

[Q15c_14] You said you were still experiencing **a change in an existing cough** after discussing it with your doctor (GP). Did you contact your doctor again after noticing that you were still experiencing the symptom?

<1>	Yes
<2>	No, but I plan to
<3>	No, and I don't plan to
<99>	Not sure
<100>	Prefer not to say

Base: all who contacted their doctor again
Question type: ***Single***
#SPD Category: health
#Question display logic:
If [Q15c_14] - Yes is selected [if Q15c_14 == 1]

[Q15d_14] You said that you contacted your doctor (GP) again after noticing that you were still experiencing **a change in an existing cough** in the last 6 months.

How long after you noticed that you were still experiencing the symptom did you contact your doctor (GP) again? If you are unsure, please give your best guess.

<1>	Within 1 week
<2>	Within 2 weeks
<3>	Within 3 weeks
<4>	Within 1 month
<5>	Within 6 weeks
<6>	Within 3 months
<7>	Within 6 months
<99>	Prefer not to say

Base: all who contacted their doctor again
Question type: ***Single***
#SPD Category: health

[Q15e_14] You said that you have experienced **a change in an existing cough** in the last 6 months. Have you had any tests or investigations to find out what is causing this symptom?

<1>	Yes
<2>	No, but I am waiting for a test
<3>	No, and I am not waiting for a test
<98>	Not sure / don't know
<99>	Prefer not to say

Base: all who had a test
*Question type: **Single***
#SPD Category: health
#Question display logic:
If [Q15e_14] - Yes is selected [if Q15e_14 == 1]

[Q15f_14] You said that you had a test or investigation for **a change in an existing cough** in the last 6 months. What were the results of your tests?

<1>	The results suggested there was nothing to worry about
<2>	The results meant I needed more tests (to investigate further)
<3>	The results meant I needed to be monitored and retested again later
<4>	The results led to a diagnosis
<5>	I have not received my test results yet
<6>	I don't remember what the test results were
<95>	Other (open [Q15f_14_open]) [open] Please specify
<99>	Prefer not to say

Base: all who had a test
*Question type: **Single***
#SPD Category: health
#Question display logic:
If [Q15f_14] - The results suggested there was nothing to worry about is selected [if Q15f_14 == 1]

[Q15g_14] Did you continue to experience the symptom after your test result said there was nothing to worry about?
Please select one answer.

<1>	Yes, I continued to experience the symptom and it was the same as before
<2>	Yes, I continued to experience the symptom, but it got better
<3>	Yes, I continued to experience the symptom and it got worse
<4>	No, the symptom went away by itself
<5>	No, the symptom went away after taking medication
<95>	Other (open [Q15g_14_open]) [open] Please specify
<99>	Prefer not to say

*Question type: **Single***
#Question display logic:
If [Q15g_14] - Yes, I continued to experience the symptom and it was the same as before or Yes, I continued to experience the symptom, but it got better or Yes, I continued to experience the symptom and it got worse or Other, is selected [if Q15g_14 in [1,2,3,95]]

[Q15h_14] You said you were still experiencing **a change in an existing cough** after receiving your test results. Did you contact your doctor again after noticing that you were still experiencing the symptom?

<1>	Yes
<2>	No, but I plan to
<3>	No, and I don’t plan to
<99>	Not sure
<100>	Prefer not to say

Question type: ***Single***
#Question display logic:
If [Q15h_14] - Yes is selected [if Q15h_14 == 1]

[Q15i_14] You said that you contacted your doctor (GP) again after noticing that you were still experiencing **a change in an existing cough** after receiving your test results.

How long after you noticed that you were still experiencing the symptom did you contact your doctor (GP) again? If you are unsure, please give your best guess.

<1>	Within 1 week
<2>	Within 2 weeks
<3>	Within 3 weeks
<4>	Within 1 month
<5>	Within 6 weeks
<6>	Within 3 months
<7>	Within 6 months
<99>	Prefer not to say

#Module display logic:
If [Q8] - Shortness of breath, Yes is selected [if Q8_15 == 1]

Question type: ***Text***

You said that you have experienced **shortness of breath** in the last 6 months. We would now like to ask you a few more questions about this.

Base: All experiencing each symptom
Question type: ***Single***
#SPD Category: health

[Q12_15] Approximately when did you first notice this symptom? Please give your best guess.
Please select one answer.

Response Option List: Q12_list

Base: All experiencing each symptom
Question type: **Single**
#SPD Category: health

[Q13_15] You said that you have experienced **shortness of breath** in the last 6 months. We would now like to ask you a few more questions about this.

How concerned have you been that this symptom might be serious?
Please select one answer.

Response Option List: Q13_list

Base: All experiencing each symptom
Question type: **Multiple**
#row order: randomize
#SPD Category: health

[Q14_15] You said that you have experienced **shortness of breath** in the last 6 months. We would now like to ask you a few more questions about this.

What do you think caused this symptom?
Please select all that apply.

Response Option List: Q14_list

Base: All experiencing each symptom
Question type: **Single**
#SPD Category: health

[Q15_15] You said that you have experienced **shortness of breath** in the last 6 months. We would now like to ask you a few more questions about this.

How long after you first noticed the symptom did you contact the GP (doctor's surgery) about it? If you are unsure, please give your best guess.
Please select one answer.

Response Option List: Q15_list

Base: All who made an appointment
Question type: ***Single***
#SPD Category: health
#Question display logic:
if Q15_15 in [3,4,5,6,7,8,9]

[Q15a_15] You said that you contacted your doctor after experiencing **shortness of breath** in the last 6 months. How long after first contacting the GP (doctor’s surgery) did the appointment take place?

This may have been an appointment that was face-to-face or remote, such as over the phone, by video call or online messaging. This also includes if a medical/health professional called you back after completing an online/e-consultation form.

If you are unsure, please give your best guess.

<1>	On the same day
<2>	On the next day
<3>	A few days later
<4>	A week later
<5>	Two weeks later
<6>	Three weeks later
<7>	Four or more weeks later
<8>	I have not had an appointment yet
<9>	Prefer not to say

Base: All who made an appointment
Question type: ***Single***
#SPD Category: health
#Question display logic:
if Q15_15 in [3,4,5,6,7,8,9] and Q15a_15 != 8

[Q15b_15] You said that you have discussed your **shortness of breath** with your doctor (GP).Did you continue to experience the symptom after you first discussed it with your doctor (GP)?

<1>	Yes, I continued to experience the symptom and it was the same as before
<2>	Yes, I continued to experience the symptom, but it got better
<3>	Yes, I continued to experience the symptom and it got worse
<4>	No, the symptom went away by itself
<5>	No, the symptom went away after taking medication
<95>	Other (open [Q15b_15_open]) [open] Please specify
<99>	Prefer not to say

Base: all who continued to experience the symptom after discussing it with their doctor
Question type: ***Single***
#SPD Category: health
#Question display logic:
If [Q15b_15] - Yes, I continued to experience the symptom and it was the same as

before or Yes, I continued to experience the symptom, but it got better or Yes, I continued to experience the symptom and it got worse or Other, is selected [if Q15b_15 in [1,2,3,95]]

[Q15c_15] You said you were still experiencing **shortness of breath** after discussing it with your doctor (GP). Did you contact your doctor again after noticing that you were still experiencing the symptom?

<1>	Yes
<2>	No, but I plan to
<3>	No, and I don't plan to
<99>	Not sure
<100>	Prefer not to say

Base: all who contacted their doctor again
*Question type: **Single***
#SPD Category: health
#Question display logic:
If [Q15c_15] - Yes is selected [if Q15c_15 == 1]

[Q15d_15] You said that you contacted your doctor (GP) again after noticing that you were still experiencing **shortness of breath** in the last 6 months.

How long after you noticed that you were still experiencing the symptom did you contact your doctor (GP) again? If you are unsure, please give your best guess.

<1>	Within 1 week
<2>	Within 2 weeks
<3>	Within 3 weeks
<4>	Within 1 month
<5>	Within 6 weeks
<6>	Within 3 months
<7>	Within 6 months
<99>	Prefer not to say

Base: all who contacted their doctor again
*Question type: **Single***
#SPD Category: health

[Q15e_15] You said that you have experienced **shortness of breath** in the last 6 months. Have you had any tests or investigations to find out what is causing this symptom?

<1>	Yes
<2>	No, but I am waiting for a test
<3>	No, and I am not waiting for a test
<98>	Not sure / don't know
<99>	Prefer not to say

Base: all who had a test
*Question type: **Single***
#SPD Category: health
#Question display logic:
If [Q15e_15] - Yes is selected [if Q15e_15 == 1]

[Q15f_15] You said that you had a test or investigation for **shortness of breath** in the last 6 months. What were the results of your tests?

<1>	The results suggested there was nothing to worry about
<2>	The results meant I needed more tests (to investigate further)
<3>	The results meant I needed to be monitored and retested again later
<4>	The results led to a diagnosis
<5>	I have not received my test results yet
<6>	I don't remember what the test results were
<95>	Other (open [Q15f_15_open]) [open] Please specify
<99>	Prefer not to say

Base: all who had a test
*Question type: **Single***
#SPD Category: health
#Question display logic:
If [Q15f_15] - The results suggested there was nothing to worry about is selected [if Q15f_15 == 1]

[Q15g_15] Did you continue to experience the symptom after your test result said there was nothing to worry about?
Please select one answer.

<1>	Yes, I continued to experience the symptom and it was the same as before
<2>	Yes, I continued to experience the symptom, but it got better
<3>	Yes, I continued to experience the symptom and it got worse
<4>	No, the symptom went away by itself
<5>	No, the symptom went away after taking medication
<95>	Other (open [Q15g_15_open]) [open] Please specify
<99>	Prefer not to say

*Question type: **Single***
#Question display logic:
If [Q15g_15] - Yes, I continued to experience the symptom and it was the same as before or Yes, I continued to experience the symptom, but it got better or Yes, I continued to experience the symptom and it got worse or Other, is selected [if Q15g_15 in [1,2,3,95]]

[Q15h_15] You said you were still experiencing **shortness of breath** after receiving your test results. Did you contact your doctor again after noticing that you were still experiencing the symptom?

<1>	Yes
<2>	No, but I plan to
<3>	No, and I don’t plan to
<99>	Not sure
<100>	Prefer not to say

---

*Question type: **Single***
#Question display logic:
If [Q15h_15] - Yes is selected [if Q15h_15 == 1]

[Q15i_15] You said that you contacted your doctor (GP) again after noticing that you were still experiencing **shortness of breath** after receiving your test results.

How long after you noticed that you were still experiencing the symptom did you contact your doctor (GP) again? If you are unsure, please give your best guess.

<1>	Within 1 week
<2>	Within 2 weeks
<3>	Within 3 weeks
<4>	Within 1 month
<5>	Within 6 weeks
<6>	Within 3 months
<7>	Within 6 months
<99>	Prefer not to say

#Module display logic:
If [Q8] - An ulcer in the mouth that doesn’t heal, Yes is selected [if Q8_16 == 1]

---

*Question type: **Text***

You said that you have experienced **an ulcer in the mouth that doesn’t heal** in the last 6 months. We would now like to ask you a few more questions about this.

Base: All experiencing each symptom
*Question type: **Single***
#SPD Category: health

[Q12_16] Approximately when did you first notice this symptom? Please give your best guess.
Please select one answer.

Response Option List: Q12_list

Base: All experiencing each symptom
Question type: ***Single***
#SPD Category: health

[Q13_16] You said that you have experienced **an ulcer in the mouth that doesn't heal** in the last 6 months. We would now like to ask you a few more questions about this.

How concerned have you been that this symptom might be serious?
Please select one answer.

Response Option List: Q13_list

Base: All experiencing each symptom
Question type: ***Multiple***
#row order: randomize
#SPD Category: health

[Q14_16] You said that you have experienced **an ulcer in the mouth that doesn't heal** in the last 6 months. We would now like to ask you a few more questions about this.

What do you think caused this symptom?
Please select all that apply.

Response Option List: Q14_list

Base: All experiencing each symptom
Question type: ***Single***
#SPD Category: health

[Q15_16] You said that you have experienced **an ulcer in the mouth that doesn't heal** in the last 6 months. We would now like to ask you a few more questions about this.

How long after you first noticed the symptom did you contact the GP (doctor's surgery) about it? If you are unsure, please give your best guess.
Please select one answer.

Response Option List: Q15_list

Base: All who made an appointment
Question type: ***Single***
#SPD Category: health
#Question display logic:
if Q15_16 in [3,4,5,6,7,8,9]

[Q15a_16] You said that you contacted your doctor after experiencing **an ulcer in the mouth that doesn't heal** in the last 6 months. How long after first contacting the GP (doctor's surgery) did the appointment take place?

This may have been an appointment that was face-to-face or remote, such as over the phone, by video call or online messaging. This also includes if a medical/health professional called you back after completing an online/e-consultation form.

If you are unsure, please give your best guess.

<1>	On the same day
<2>	On the next day
<3>	A few days later
<4>	A week later
<5>	Two weeks later
<6>	Three weeks later
<7>	Four or more weeks later
<8>	I have not had an appointment yet
<9>	Prefer not to say

Base: All who made an appointment
Question type: ***Single***
#SPD Category: health
#Question display logic:
if Q15_16 in [3,4,5,6,7,8,9] and Q15a_16 != 8

[Q15b_16] You said that you have discussed **an ulcer in the mouth that doesn't heal** with your doctor (GP). Did you continue to experience the symptom after you first discussed it with your doctor (GP)?

<1>	Yes, I continued to experience the symptom and it was the same as before
<2>	Yes, I continued to experience the symptom, but it got better
<3>	Yes, I continued to experience the symptom and it got worse
<4>	No, the symptom went away by itself
<5>	No, the symptom went away after taking medication
<95>	Other (open [Q15b_16_open]) [open] Please specify
<99>	Prefer not to say

Base: all who continued to experience the symptom after discussing it with their doctor
Question type: ***Single***
#SPD Category: health
#Question display logic:
If [Q15b_16] - Yes, I continued to experience the symptom and it was the same as

before or Yes, I continued to experience the symptom, but it got better or Yes, I continued to experience the symptom and it got worse or Other, is selected [if Q15b_16 in [1,2,3,95]]

[Q15c_16] You said you were still experiencing **an ulcer in the mouth that doesn't heal** after discussing it with your doctor (GP). Did you contact your doctor again after noticing that you were still experiencing the symptom?

<1>	Yes
<2>	No, but I plan to
<3>	No, and I don't plan to
<99>	Not sure
<100>	Prefer not to say

Base: all who contacted their doctor again
*Question type: **Single***
#SPD Category: health
#Question display logic:
If [Q15c_16] - Yes is selected [if Q15c_16 == 1]

[Q15d_16] You said that you contacted your doctor (GP) again after noticing that you were still experiencing **an ulcer in the mouth that doesn't heal** in the last 6 months.

How long after you noticed that you were still experiencing the symptom did you contact your doctor (GP) again? If you are unsure, please give your best guess.

<1>	Within 1 week
<2>	Within 2 weeks
<3>	Within 3 weeks
<4>	Within 1 month
<5>	Within 6 weeks
<6>	Within 3 months
<7>	Within 6 months
<99>	Prefer not to say

Base: all who contacted their doctor again
*Question type: **Single***
#SPD Category: health

[Q15e_16] You said that you have experienced **an ulcer in the mouth that doesn't heal** in the last 6 months. Have you had any tests or investigations to find out what is causing this symptom?

<1>	Yes
<2>	No, but I am waiting for a test
<3>	No, and I am not waiting for a test
<98>	Not sure / don't know
<99>	Prefer not to say

Base: all who had a test
Question type: ***Single***
#SPD Category: health
#Question display logic:
If [Q15e_16] - Yes is selected [if Q15e_16 == 1]

[Q15f_16] You said that you had a test or investigation for **an ulcer in the mouth that doesn't heal** in the last 6 months. What were the results of your tests?

<1>	The results suggested there was nothing to worry about
<2>	The results meant I needed more tests (to investigate further)
<3>	The results meant I needed to be monitored and retested again later
<4>	The results led to a diagnosis
<5>	I have not received my test results yet
<6>	I don't remember what the test results were
<95>	Other (open [Q15f_16_open]) [open] Please specify
<99>	Prefer not to say

Base: all who had a test
Question type: ***Single***
#SPD Category: health
#Question display logic:
If [Q15f_16] - The results suggested there was nothing to worry about is selected [if Q15f_16 == 1]

[Q15g_16] Did you continue to experience the symptom after your test result said there was nothing to worry about?
Please select one answer.

<1>	Yes, I continued to experience the symptom and it was the same as before
<2>	Yes, I continued to experience the symptom, but it got better
<3>	Yes, I continued to experience the symptom and it got worse
<4>	No, the symptom went away by itself
<5>	No, the symptom went away after taking medication
<95>	Other (open [Q15g_16_open]) [open] Please specify
<99>	Prefer not to say

Question type: ***Single***
#Question display logic:
If [Q15g_16] - Yes, I continued to experience the symptom and it was the same as before or Yes, I continued to experience the symptom, but it got better or Yes, I continued to experience the symptom and it got worse or Other, is selected [if Q15g_16 in [1,2,3,95]]

[Q15h_16] You said you were still experiencing **an ulcer in the mouth that doesn't heal** after receiving your test results. Did you contact your doctor again after noticing that you were still experiencing the symptom?

<1>	Yes
<2>	No, but I plan to
<3>	No, and I don't plan to
<99>	Not sure
<100>	Prefer not to say

Question type: ***Single***
#Question display logic:
If [Q15h_16] - Yes is selected [if Q15h_16 == 1]

[Q15i_16] You said that you contacted your doctor (GP) again after noticing that you were still experiencing **an ulcer in the mouth that doesn't heal** after receiving your test results.

How long after you noticed that you were still experiencing the symptom did you contact your doctor (GP) again? If you are unsure, please give your best guess.

<1>	Within 1 week
<2>	Within 2 weeks
<3>	Within 3 weeks
<4>	Within 1 month
<5>	Within 6 weeks
<6>	Within 3 months
<7>	Within 6 months
<99>	Prefer not to say

#Module display logic:
If [Q8] - Red or white patches in your mouth, Yes is selected [if Q8_17 == 1]

Question type: ***Text***

You said that you have experienced **red or white patches in your mouth** in the last 6 months. We would now like to ask you a few more questions about this.

Base: All experiencing each symptom
Question type: ***Single***
#SPD Category: health

[Q12_17] Approximately when did you first notice this symptom? Please give your best guess.
Please select one answer.

Response Option List: Q12_list

Base: All experiencing each symptom
*Question type: **Single***
#SPD Category: health

[Q13_17] You said that you have experienced **red or white patches in your mouth** in the last 6 months. We would now like to ask you a few more questions about this.

How concerned have you been that this symptom might be serious?
Please select one answer.

Response Option List: Q13_list

Base: All experiencing each symptom
*Question type: **Multiple***
#row order: randomize
#SPD Category: health

[Q14_17] You said that you have experienced **red or white patches in your mouth** in the last 6 months. We would now like to ask you a few more questions about this.

What do you think caused this symptom?
Please select all that apply.

Response Option List: Q14_list

Base: All experiencing each symptom
*Question type: **Single***
#SPD Category: health

[Q15_17] You said that you have experienced **red or white patches in your mouth** in the last 6 months. We would now like to ask you a few more questions about this.

How long after you first noticed the symptom did you contact the GP (doctor's surgery) about it? If you are unsure, please give your best guess.
Please select one answer.

Response Option List: Q15_list

Base: All who made an appointment
*Question type: **Single***
#SPD Category: health
#Question display logic:
if Q15_17 in [3,4,5,6,7,8,9]

[Q15a_17] You said that you contacted your doctor after experiencing **red or white patches in your mouth** in the last 6 months. How long after first contacting the GP (doctor's surgery) did the appointment take place?

This may have been an appointment that was face-to-face or remote, such as over the phone, by video call or online messaging. This also includes if a medical/health professional called you back after completing an online/e-consultation form.

If you are unsure, please give your best guess.

<1>	On the same day
<2>	On the next day
<3>	A few days later
<4>	A week later
<5>	Two weeks later
<6>	Three weeks later
<7>	Four or more weeks later
<8>	I have not had an appointment yet
<9>	Prefer not to say

Base: All who made an appointment
*Question type: **Single***
#SPD Category: health
#Question display logic:
if Q15_17 in [3,4,5,6,7,8,9] and Q15a_17 != 8

[Q15b_17] You said that you have discussed the **red or white patches in your mouth** with your doctor (GP). Did you continue to experience the symptom after you first discussed it with your doctor (GP)?

<1>	Yes, I continued to experience the symptom and it was the same as before
<2>	Yes, I continued to experience the symptom, but it got better
<3>	Yes, I continued to experience the symptom and it got worse
<4>	No, the symptom went away by itself
<5>	No, the symptom went away after taking medication
<95>	Other (open [Q15b_17_open]) [open] Please specify
<99>	Prefer not to say

Base: all who continued to experience the symptom after discussing it with their doctor
*Question type: **Single***
#SPD Category: health
#Question display logic:
If [Q15b_17] - Yes, I continued to experience the symptom and it was the same as

before or Yes, I continued to experience the symptom, but it got better or Yes, I continued to experience the symptom and it got worse or Other, is selected [if Q15b_17 in [1,2,3,95]]

[Q15c_17] You said you were still experiencing **red or white patches in your mouth** after discussing it with your doctor (GP). Did you contact your doctor again after noticing that you were still experiencing the symptom?

<1>	Yes
<2>	No, but I plan to
<3>	No, and I don’t plan to
<99>	Not sure
<100>	Prefer not to say

Base: all who contacted their doctor again
Question type: ***Single***
#SPD Category: health
#Question display logic:
If [Q15c_17] - Yes is selected [if Q15c_17 == 1]

[Q15d_17] You said that you contacted your doctor (GP) again after noticing that you were still experiencing **red or white patches in your mouth** in the last 6 months.

How long after you noticed that you were still experiencing the symptom did you contact your doctor (GP) again? If you are unsure, please give your best guess.

<1>	Within 1 week
<2>	Within 2 weeks
<3>	Within 3 weeks
<4>	Within 1 month
<5>	Within 6 weeks
<6>	Within 3 months
<7>	Within 6 months
<99>	Prefer not to say

Base: all who contacted their doctor again
Question type: ***Single***
#SPD Category: health

[Q15e_17] You said that you have experienced **red or white patches in your mouth** in the last 6 months. Have you had any tests or investigations to find out what is causing this symptom?

<1>	Yes
<2>	No, but I am waiting for a test
<3>	No, and I am not waiting for a test
<98>	Not sure / don’t know
<99>	Prefer not to say

Base: all who had a test
*Question type: **Single***
#SPD Category: health
#Question display logic:
If [Q15e_17] - Yes is selected [if Q15e_17 == 1]

[Q15f_17] You said that you had a test or investigation **red or white patches in your mouth** in the last 6 months. What were the results of your tests?

<1>	The results suggested there was nothing to worry about
<2>	The results meant I needed more tests (to investigate further)
<3>	The results meant I needed to be monitored and retested again later
<4>	The results led to a diagnosis
<5>	I have not received my test results yet
<6>	I don't remember what the test results were
<95>	Other (open [Q15f_17_open]) [open] Please specify
<99>	Prefer not to say

Base: all who had a test
*Question type: **Single***
#SPD Category: health
#Question display logic:
If [Q15f_17] - The results suggested there was nothing to worry about is selected [if Q15f_17 == 1]

[Q15g_17] Did you continue to experience the symptom after your test result said there was nothing to worry about?
Please select one answer.

<1>	Yes, I continued to experience the symptom and it was the same as before
<2>	Yes, I continued to experience the symptom, but it got better
<3>	Yes, I continued to experience the symptom and it got worse
<4>	No, the symptom went away by itself
<5>	No, the symptom went away after taking medication
<95>	Other (open [Q15g_17_open]) [open] Please specify
<99>	Prefer not to say

*Question type: **Single***
#Question display logic:
If [Q15g_17] - Yes, I continued to experience the symptom and it was the same as before or Yes, I continued to experience the symptom, but it got better or Yes, I continued to experience the symptom and it got worse or Other, is selected [if Q15g_17 in [1,2,3,95]]

[Q15h_17] You said you were still experiencing **red or white patches in your mouth** after receiving your test results. Did you contact your doctor again after noticing that you were still experiencing the symptom?

<1>	Yes
<2>	No, but I plan to
<3>	No, and I don’t plan to
<99>	Not sure
<100>	Prefer not to say

Question type: ***Single***
#Question display logic:
If [Q15h_17] - Yes is selected [if Q15h_17 == 1]

[Q15i_17] You said that you contacted your doctor (GP) again after noticing that you were still experiencing **red or white patches in your mouth** after receiving your test results.

How long after you noticed that you were still experiencing the symptom did you contact your doctor (GP) again? If you are unsure, please give your best guess.

<1>	Within 1 week
<2>	Within 2 weeks
<3>	Within 3 weeks
<4>	Within 1 month
<5>	Within 6 weeks
<6>	Within 3 months
<7>	Within 6 months
<99>	Prefer not to say

Question type: ***Text***

We would now like to ask a few questions about any recent medical attention you may have sought. You may have already answered some similar questions earlier in the survey about a specific symptom or health problem. For the following questions, please think about all symptoms that you have experienced including the ones you have discussed before and any others.

Question type: ***Text***

We would now like to ask a few questions about any recent medical attention you may have sought. You may have already answered some similar questions earlier in the survey about a specific symptom or health problem. For the following questions, please think about all symptoms that you have experienced including the ones you have discussed before and any others.

Base: All
Question type: ***Single***

[Q17] In the last 6 months, have you considered contacting, or did you try to contact your GP practice to discuss your own health concern with a medical/health professional (e.g. a doctor/GP or nurse)?

You may have tried to contact your GP practice in different ways, including calling them or using an online form.

Please select one answer.

<1>	Yes, I considered contacting my GP practice, but did not contact them
<2>	Yes, I contacted or tried to contact my GP practice
<3>	No, I did not consider contacting or try to contact my GP practice
<4>	I don't remember
<5>	Prefer not to say

Base: All who considered but did not contact GP
Question type: ***Multiple***

[Q18] You said that in the last 6 months you have considered contacting your GP practice to discuss a health concern with a medical/health professional but did not contact them. Which, if any, of the following happened next?
Please select all that apply.

<1>	I did not speak to a medical/health professional about my health concern
<2>	I spoke to a pharmacist about my health concern
<3>	I called NHS 111
<9>	I went to A&E
<4>	I spoke to a family member or friend about my health concern
<5>	I looked for information about my health concern somewhere else e.g. online
<6 fixed>	Other (open [Q18_6_open]) [open]
<7 fixed xor>	I don't remember
<8 fixed xor>	Prefer not to say

Base: All who tried to contact GP
Question type: ***Single***
#Question display logic:
If [Q17] - Yes, I tried to contact my GP practice is selected [if Q17 == 2]

[Q19] You said that in the last 6 months you contacted or tried to contact your GP practice to discuss a health concern with a medical/health professional. Which, if any, of the following best describes what happened next?
You may have tried to contact your GP practice in different ways, including calling them or using an online form.

<1>	I made an appointment with a medical/health professional the first time I tried to contact my GP practice (The appointment may have been face-to-face or remote. A remote appointment can have been over the phone, video call or online messaging, or if a doctor or other health professional called you back)
<2>	I made an appointment with a medical/health professional after trying to contact my GP practice more than once (The appointment may have been face-to-face or remote. A remote appointment can have been over the phone, video call or online messaging, or if a doctor or other health professional called you back)
<3>	I tried to contact my GP practice but could not get through/didn't get a response/wasn't called back
<4>	I tried to contact my GP practice but could not get an appointment
<5 fixed>	Other (open [Q19_5_open]) [open]
<6 fixed>	I don't remember
<7 fixed>	Prefer not to say

Base: All who made an appointment/other/refused
Question type: ***Single***
#Question display logic:
If [Q19] - I made an appointment with a medical/health professional the first time I tried to contact my GP practice (The appointment may have been face-to-face or remote. A remote appointment can have been over the phone, video call or online messaging, or if a doctor or other health professional called you back) or I made an appointment with a medical/health professional after trying to contact my GP practice more than once (The appointment may have been face-to-face or remote. A remote appointment can have been over the phone, video call or online messaging, or if a doctor or other health professional called you back) or Other or Prefer not to say, is selected [if Q19 in [1,2,5,7]]

[Q20] Which of the following best describes the type of appointment you had?
Please select one answer.

<1>	I saw a medical/health professional in person at my GP practice
<2>	I spoke to a medical/health professional on the phone (you could hear each other only)
<3>	I spoke to a medical/health professional on a video call (you could hear and see each other)
<4>	I spoke to a medical/health professional using online messaging (for example, by online form, email or smartphone App)
<5>	Other (open [Q20_5_open]) [open]
<6 fixed>	I have not had an appointment with my GP practice in the last 6 months
<7 fixed>	I don't remember
<8 fixed>	Prefer not to say

Base: All who had a remote appointment/other/refused
Question type: ***Grid***
#row order: randomize
#Question display logic:
If [Q20] - I spoke to a medical/health professional on the phone (you could hear each

other only) or I spoke to a medical/health professional on a video call (you could hear and see each other) or I spoke to a medical/health professional using online messaging (for example, by online form, email or smartphone App) or Other or Prefer not to say, is selected [if Q20 in [2,3,4,5,8]]

[Q24] If you had a remote consultation/appointment with a medical/health professional from your GP practice in the last 6 months, please tell us to what extent you agree or disagree with the following statements.
A remote consultation/appointment is one where you did not see the medical/health professional in person. The remote consultation/appointment may have taken place over the phone, using video call or using online messaging (for example, by email or smartphone App). A remote appointment/consultation also includes if a medical/health professional called you.
Please select one answer per statement.

-[Q24_2]	Remote GP consultations make me feel safer from COVID-19 compared with attending face to face
-[Q24_3]	I am concerned that remote GP consultations may result in the wrong decision being made about my care
-[Q24_6]	I felt comfortable discussing my health concern via remote GP consultation
-[Q24_7]	Remote GP consultation allowed my concerns to be adequately addressed
-[Q24_10]	The remote GP consultation was not helpful because I needed to see the doctor in person anyway
<1>	Strongly agree
<2>	Somewhat agree
<3>	Somewhat disagree
<4>	Strongly disagree
<5>	Not applicable/I have not had a remote consultation in the last 6 months
<6>	Prefer not to say

Base: All
*Question type: **Multiple***
#row order: randomize
#SPD Category: health

[Q25] The last time you saw or spoke to a medical/health professional about your health, which, if any, of the following played a role in your decision to do so?
(This may have been an appointment with a medical/health professional (e.g. a doctor, nurse or pharmacist) in person, online or over the phone).
Please select all that apply.

<1>	I had a symptom that I thought might be a sign of cancer	<10>	I had seen information about this symptom in the media (e.g. on tv, radio, posters or magazines)
<2>	I had a symptom that was unusual for me	<11>	I could have a remote consultation (for example, by phone, email or video call)
<3>	I had a symptom that was painful	<12>	I was attending an appointment for an existing problem/condition
<4>	I knew someone who had a similar symptom, and it turned out to be serious	<13>	I had a symptom that was getting worse

<5>	I had a symptom that didn’t go away	<14 fixed>	Other (open [Q25_14_open]) [open]
<6>	My friends or family encouraged me to go	<15 fixed xor>	I have never sought medical attention
<7>	I had a symptom, but I didn’t know what was causing it	<16 fixed xor>	I don’t remember
<8>	I had a symptom that was “bothersome”	<17 fixed xor>	Prefer not to say
<9>	I had a feeling that something wasn’t right		

Base: All
*Question type: **Multiple***
#row order: randomize #Columns: 2

[Q26] Thinking about the last time you considered seeing or speaking to a medical/health professional about your health, which, if any, of the following put you off, or made you delay doing so?
(This may have been an appointment with a medical/health professional (e.g. a doctor, nurse or pharmacist) in person, online or over the phone).
Please select all that apply.

<1>	I found it embarrassing talking about my symptoms	<14>	I worried about catching COVID-19
<2>	I worried about wasting the healthcare professional’s time	<15>	I worried about putting extra strain on the NHS / health services
<3>	I found it difficult to get an appointment with a particular health professional	<16>	I had symptoms that might have been related to COVID-19
<4>	I found it difficult to get an appointment at a convenient time	<17>	I expected to be given a remote consultation and would have been difficult for me to discuss my health problem remotely (by phone, email or video call)
<5>	I was too busy to make time to seek medical attention	<24>	I could not afford to cover the costs related to having an appointment (e.g. transport, childcare, reduced pay/earnings)
<6>	I had too many other things to worry about	<25>	I worried my pay/earnings would be affected if I needed to have further tests or treatment
<7>	I worried about what they might find wrong with me	<18>	I found it difficult to get an appointment
<8>	I didn’t feel confident talking about my symptom(s)	<19>	I thought the symptom was related to an existing illness or condition
<9>	I worried they wouldn’t take my symptom(s) seriously	<20 fixed>	Other (open [Q26_20_open]) [open]
<10>	I didn’t want to be seen as someone who makes a fuss	<21 fixed xor>	Nothing put me off/delayed me in seeking medical attention

<11>	I didn't want to talk to a receptionist/administrative person about my symptom(s)	<22 fixed xor>	I don't remember
<12>	I worried about the possibility of having treatment	<23 fixed xor>	Prefer not to say
<13>	I worried about the impact on my employment from taking time off		

Base: All who put off/delayed speaking to a health professional and selected more than one option from codes 1-20 at Q26

#scripting note if respondents select only one code between 1-20 at Q26, automatically code this at Q26b without showing them the question
Question type: ***Rank***
#order: randomize

Show only response options that were selected in [Q26]

[Q26b] You said that you put off or delayed seeing or speaking to a health professional for the following reasons…

Which ONE reason had the greatest impact on you putting off or delaying seeing or speaking to a health professional? Please select one answer only.

<1>	I found it embarrassing talking about my symptoms	<14>	I worried about catching COVID-19
<2>	I worried about wasting the healthcare professional's time	<15>	I worried about putting extra strain on the NHS / health services
<3>	I found it difficult to get an appointment with a particular health professional	<16>	I had symptoms that might have been related to COVID-19
<4>	I found it difficult to get an appointment at a convenient time	<17>	I expected to be given a remote consultation and would have been difficult for me to discuss my health problem remotely (by phone, email or video call)
<5>	I was too busy to make time to seek medical attention	<24>	I could not afford to cover the costs related to having an appointment (e.g. transport, childcare, reduced pay/earnings)
<6>	I had too many other things to worry about	<25>	I worried my pay/earnings would be affected if I needed to have further tests or treatment
<7>	I worried about what they might find wrong with me	<18>	I found it difficult to get an appointment
<8>	I didn't feel confident talking about my symptom(s)	<19>	I thought the symptom was related to an existing illness or condition

<9>	I worried they wouldn't take my symptom(s) seriously	<20 fixed>	Other
<10>	I didn't want to be seen as someone who makes a fuss	<21>	Don't know
<11>	I didn't want to talk to a receptionist/administrative person about my symptom(s)	<22>	Prefer not to say
<12>	I worried about the possibility of having treatment		
<13>	I worried about the impact on my employment from taking time off		

Base: All
Question type: ***Grid***
#row order: randomize

[Q27] To what extent do you agree or disagree with the following statements.
Please select one answer per statement

-[Q27_1]	I am confident that I would be safe from COVID-19 if I needed to attend an appointment at my GP surgery.
-[Q27_6]	I am confident I would be safe from COVID-19 if I needed to attend a medical appointment on a mobile unit (by 'mobile unit' we mean a small medical van or trailer which can be moved to provide medical care or testing in different locations)
-[Q27_7]	I think a remote consultation counts as an official consultation with my doctor. (A remote consultation is one where you do not see the medical/health professional in person. Remote consultations/appointments may take place over the phone, using video call or using online messaging)
-[Q27_8]	I think a remote consultation is as useful as a consultation in person. (A remote consultation is one where you do not see the medical/health professional in person. Remote consultations/appointments may take place over the phone, using video call or using online messaging)
<1>	Strongly agree
<2>	Somewhat agree
<3>	Somewhat disagree
<4>	Strongly disagree
<5>	I don't know
<6>	Prefer not to say

Base: all who did not contact a GP about a health problem
Question type: ***Single***

[Q27a] In the past **6 months**, have you contacted your doctor (GP) about any symptoms or health problems that you were experiencing?

<1>	Yes
<2>	No
<99>	Prefer not to say

Question type: ***Text***

The next few questions are about contacting your doctor (GP) about a symptom or health problem. You may have already answered some similar questions earlier in the survey about a specific symptom or health problem. For the following questions, please think about all symptoms that you have experienced including the ones you have discussed before and any others.

#Question display logic:
if Q27a in [1,3] or prior_GP==1

Question type: ***Single***

[Q27b] In the last 6 months, have you gone back to your doctor (GP) with the same symptom or health problem because you were still experiencing it?

<1>	Yes, as I was still experiencing the symptom and it was the same as before
<2>	Yes, as I was still experiencing the symptom, but it had got better
<3>	Yes, as I was still experiencing the symptom and it had got worse
<4>	No, but I was still experiencing the symptom
<5>	No, as I had a management plan in place for the symptom
<6>	No, as the symptom went away by itself
<7>	No, as the symptom went away after taking medication
<8>	Other (open [Q27b_other]) [open] please specify
<10>	Not relevant to me
<9>	I don't remember
<99>	Prefer not to say

Question type: ***Single***
#SPD Category: health

[Q27d] Have you had any tests for this symptom?

<1>	Yes
<2>	No, but I am waiting for a test
<3>	No, and I am not waiting for a test
<98>	Not sure / don't know

Base: all who had a test
*Question type: **Single***
#SPD Category: health
#Question display logic:
If [Q27d] - Yes is selected [if Q27d == 1]

[Q27e] What were the results of your tests?

<1>	The results suggested there was nothing to worry about
<2>	The results meant I needed more tests (to investigate further)
<3>	The results meant I needed to be monitored and retested again later
<4>	The results led to a diagnosis
<5>	I have not received my test results yet
<6>	I don't remember what the test results were
<95>	Other (open [Q27e_open]) [open] Please specify
<99>	Prefer not to say

Base: all who have contacted a GP in the past 6 months
*Question type: **Single***
#Question display logic:
If [Q27e] - The results suggested there was nothing to worry about is selected [if Q27e == 1]

[Q27c] In the last 6 months, after a test result suggested there was nothing to worry about, have you gone back to your doctor (GP) with the same symptom or health problem because you were still experiencing it?
Please select one answer.

<1>	Yes, as I was still experiencing the symptom and it was the same as before
<2>	Yes, as I was still experiencing the symptom, but it had got better
<3>	Yes, as I was still experiencing the symptom and it had got worse
<4>	No, but I was still experiencing the symptom
<5>	No, as I had a management plan in place for the symptom
<6>	No, as the symptom went away by itself
<7>	No, as the symptom went away after taking medication
<8>	Other (open [Q27c_other]) [open] please specify
<95>	I don't remember
<99>	Prefer not to say

Base: All
*Question type: **Multiple***
#row order: randomize #Columns: 2

[Q29] If your doctor (GP) asked you to go for a test at a hospital, would any of the following make you more likely to go?

Please select all that apply.

<1> If I could choose/change the day/time of the test
<2> If the hospital gave me a specific day/time to go for the test
<3> If I had to arrange the day/time of the test myself
<4> If I was given more notice of the appointment day/time of the test
<5> If it was easier to take time off work to go for the test
<6> If it was easier to get to the hospital (e.g. transport was provided for me or free parking)
<7> If the test was done by a person that is the same sex as me
<8> If I could take someone with me
<9> If I could do the test at home instead
<10> If I could get someone to cover my childcare or caring responsibilities
<11> If I was given more information about what the test involved
<12> If I was able to speak to others who have had the test
<13> If I received reminders about my appointment e.g. by text or email
<14> If there were processes in place to reduce the spread of coronavirus (e.g. social distancing, masks to be worn, one-way systems)
<15 fixed> Other [Q29_15_open] {open prompt=""}
<16 fixed xor> Nothing would make me more likely to go
<17 fixed xor> Prefer not to say

Base: All
*Question type: **Grid***
#row order: randomize

[Q30] To what extent do you agree or disagree with the following statements.
Please select one answer per statement

-[Q30_1]	I am confident that I would be safe from COVID-19 if I needed to attend an appointment at a hospital.
-[Q30_4_2]	I don't think the health service has enough staff or equipment to see, test and treat all the people with cancer that need to be seen, tested and treated
<1>	Strongly agree
<2>	Somewhat agree
<3>	Somewhat disagree
<4>	Strongly disagree
<6>	I don't know
<7>	Prefer not to say

---

*Question type: **Text***

Next, we would like to ask a few questions about your awareness of cancer. Please note this is not a test and we are simply trying to understand your current awareness or beliefs about this disease.

Base: All
*Question type: **Multiple***

[Q31] Please list as many warning signs and symptoms of cancer as you can think of in the boxes below:
Please type one answer in each box

<1>	undefined (open [Q31_1open]) [open]	<8>	undefined (open [Q31_8open]) [open]
<2>	undefined (open [Q31_2open]) [open]	<9>	undefined (open [Q31_9open]) [open]
<3>	undefined (open [Q31_3open]) [open]	<10>	undefined (open [Q31_10open]) [open]
<4>	undefined (open [Q31_4open]) [open]	<11>	undefined (open [Q31_11open]) [open]
<5>	undefined (open [Q31_5open]) [open]	<12>	undefined (open [Q31_12open]) [open]
<6>	undefined (open [Q31_6open]) [open]	<13 fixed xor>	Not sure
<7>	undefined (open [Q31_7open]) [open]		

Base: All
*Question type: **Grid***
#row order: randomize

[Q32] Which of the following, if any, do you think could be warning signs or symptoms of cancer?
You may have already mentioned some of these in the last question.
Please select one answer per symptom.

-[Q32_1]	An unexplained lump or swelling	-[Q32_9]	A sore that does not heal
-[Q32_2]	Persistent unexplained pain (persistent means doesn't go away)	-[Q32_10]	Unexplained weight loss
-[Q32_3]	Unexplained bleeding	-[Q32_11]	Persistent hoarseness (persistent means doesn't go away)
-[Q32_4]	A persistent cough (persistent means doesn't go away)	-[Q32_12]	Coughing up blood
-[Q32_5]	A persistent change in bowel habits (persistent means doesn't go away)	-[Q32_13]	Shortness of breath
-[Q32_6]	A persistent change in bladder habits (persistent means doesn't go away)	-[Q32_14]	Feeling tired all the time
-[Q32_7]	A persistent difficulty swallowing (persistent means doesn't go away)	-[Q32_15]	A change to an existing cough
-[Q32_8]	A change in the appearance of a mole		

<1> **Yes, I think this could be a sign of cancer**
<2> **No, I don't think this could be a sign of cancer**
<3> **Don't know/ not sure**

Base: All
*Question type: **Multiple***

[Q33] What things do you think could increase a person's chance of developing cancer? Please list as many things you can think of in the boxes below.
Please type one answer in each box

<1>	undefined (open [Q31_1_open]) [open]	<8>	undefined (open [Q31_8_open]) [open]
<2>	undefined (open [Q31_2_open]) [open]	<9>	undefined (open [Q31_9_open]) [open]
<3>	undefined (open [Q31_3_open]) [open]	<10>	undefined (open [Q31_10_open]) [open]
<4>	undefined (open [Q31_4_open]) [open]	<11>	undefined (open [Q31_11_open]) [open]
<5>	undefined (open [Q31_5_open]) [open]	<12>	undefined (open [Q31_12_open]) [open]
<6>	undefined (open [Q31_6_open]) [open]	<13 fixed xor>	Not sure
<7>	undefined (open [Q31_7_open]) [open]		

Base: All
Question type: ***Grid***
#row order: randomize

[Q34] Which of the following, if any, do you think could increase a person's chance of developing cancer?
You may have already mentioned some of these in the last question.
Please select one answer per option

-[Q34_1]	Using mobile phones	-[Q34_9]	Getting sunburnt
-[Q34_2]	Feeling stressed	-[Q34_10]	Eating processed meat (eg. bacon, ham, salami, corned beef, sausage)
-[Q34_3]	Smoking	-[Q34_11]	Exposure to another person's smoking
-[Q34_4]	Being overweight	-[Q34_12]	Being older
-[Q34_5]	Being obese	-[Q34_13]	Infection with HPV (HPV - a type of virus that infects the skin and the cells lining body cavities)
-[Q34_6]	Having a close relative with cancer	-[Q34_14]	Not eating enough fibre
-[Q34_7]	Drinking alcohol	-[Q34_15]	Having a previous history of lung disease, such as, Chronic Obstructive Pulmonary Disease (COPD)
-[Q34_8]	Not doing enough physical activity	[Q34_16]	Using e-cigarettes/vapes

<1>	**Yes, I think this could increase a person's chance of developing cancer**
<2>	**No, I don't think this could increase a person's chance of developing cancer**
<3>	**Don't know / Not sure**

Question type: **Text**

Please note: Mobile phone usage and stress are not risk factors, and do not increase a person's chance of developing cancer.

E-cigarettes are far less harmful than smoking and can help people who smoke to stop. There is currently no good evidence that e-cigarettes cause cancer. But e-cigarettes are not risk-free. We don't yet know their long-term effects, so children and people who have never smoked shouldn't use them.

Every other risk factor from the previous question could increase a person's chance of developing cancer. The list is not exhaustive of risk factors of developing cancer.

Base: All
Question type: **Multiple**
#SPD Category: health

[Q35] Have you, anyone in your family or any of your friends had cancer?
Please select all that apply.

<1>	Me
<2>	My partner
<3>	Someone in my immediate family (e.g. parents, grandparents, siblings)
<4>	Someone in my wider family (e.g. cousin, aunt, uncle)
<5>	A close friend
<6>	An acquaintance
<7>	A colleague
<8 fixed>	Someone else (open [Q35_8_open]) [open]
<9 fixed xor>	None of those
<10 fixed xor>	Prefer not to say

Base: All
Question type: **Single**

[S1] What is your sex?

A later question gives the option to tell us if your gender is different from your sex registered at birth, and, if different, to record your gender.

We are asking this question because certain cancers, and some other health matters covered in this survey, are affected by your sex.

<1> Male
<2> Female
<3> Prefer not to say

Base: All
*Question type: **Single***

[S2] Is your gender the same as the sex you were registered at birth?

<1> Yes
<2> No (open [S2_other]) [open] please write in gender
<3> Prefer not to say

---

*Question type: **Pdl***
#Question display logic:
if 0
[Varlabel - Age]

[age] What is your age?

Range: 0 ~ 120

---

*Question type: **Text***

The next questions are about cervical screening. Cervical screening is offered to women, some transgender men and some non-binary people aged 25 to 64 in the UK. It is sometimes called a smear test, Pap test or HPV test.

Base: Female/refused and 25+
*Question type: **Single***
#Question display logic:
if S1 in [2,3] and (age_test > 24)

[Q36] Did you go for cervical screening the last time you were invited?
Please select one answer.

<1> Yes
<2> No

<3>	I have never been invited
<4>	I am not eligible
<5>	Don’t know
<6>	Prefer not to say

Base: Female/refused and 25+
Question type: ***Single***
#Question display logic:
if S1 in [2,3] and (age_test > 24)

[Q37] Will you go for cervical screening next time you are invited?
Please select one answer.

<1>	Yes, definitely
<2>	Yes, probably
<3>	No, probably not
<4>	No, definitely not
<5>	I’m not eligible to be invited in the future
<6>	Don’t know
<7>	Prefer not to say

Base: Those who have been invited/DK/refused
Question type: ***Multiple***
#row order: randomize
#Question display logic:
If [Q36] - Yes or No or Don’t know or Prefer not to say, is selected [if Q36 in [1,2,5,6]]

[Q38] Thinking about the last time you were invited for cervical screening, did any of the following put you off going?
Please select all that apply.

<1>	I was worried that cervical screening might be painful	<14>	I found it difficult to get an appointment
<2>	I didn’t have any symptoms of cervical cancer	<15>	I worried about putting extra strain on the NHS / health services
<3>	I had other more important things to worry about than cervical screening	<22>	After thinking about cervical screening, I decided that the harms of taking part outweigh the benefits
<4>	I was too busy to go for cervical screening	<16>	I have found cervical screening painful when I have been before
<5>	I don’t think that I am at risk of cervical cancer	<23>	I could not afford to cover the costs related to having an appointment (e.g. transport, childcare, reduced pay/earnings)
<6>	I was too embarrassed to go for cervical screening	<24>	I worried my pay/earnings would be affected if I needed

			to have further tests or treatment
<7>	I was too frightened of what the test might find	<17 fixed>	Other (open [Q38_17_open]) [open]
<8>	I didn't want a man to carry out the screening test	<18 fixed xor>	I have never been invited for cervical screening
<9>	I have had a bad experience of cervical screening in the past	<19 fixed xor>	Prefer not to say
<11>	I was too afraid of having treatment if I was found to have cancer	<20 fixed xor>	Nothing put me off going
<12>	I was worried about catching COVID-19 if I went for screening	<21 fixed xor>	I don't remember
<13>	I had symptoms that might have been related to COVID-19		

---

*Question type: **Text***

Please note: Screening is for people who don't have symptoms. If you have symptoms, please speak to your GP.

#Question display logic:
if S1 in [2,3] and (age_test < 65)

#Question display logic:
if split_number==1

---

*Question type: **Pdl***
#Question display logic:
if is_test and updated
[Varlabel - Region lived]

[profile_GOR] UK regions and countries lived in [calculated from postcode]

<1>	North East	<8>	South East
<2>	North West	<9>	South West
<3>	Yorkshire and the Humber	<10>	Wales
<4>	East Midlands	<11>	Scotland
<5>	West Midlands	<12>	Northern Ireland
<6>	East of England	<13 if 0>	Non UK & Invalid
<7>	London		

Question type: ***Text***

The next questions are about bowel cancer screening, where a poo test kit is sent to you to do at home. This used to be known as the Faecal Occult Blood (FOB) Test, but is now known as the Faecal Immunochemical Test (FIT).

Base: Eligible for bowel screening based on age/nation (50 and over in Eng, Scot and Wales; 60 and over in NI)
Question type: ***Single***
#Question display logic:
if (age_test > 59 and profile_GOR==12) or (age_test > 49 and profile_GOR in [1,2,3,4,5,6,7,8,9,10,11])

[Q40] Did you complete a bowel cancer screening poo test kit last time you were sent one? (Faecal occult blood (FOB) test, or Faecal Immunochemical Test (FIT)
Please select one answer.

<1>	Yes
<2>	No
<3>	I have never been sent a kit
<4>	I am not eligible
<5>	Don’t know
<6>	Prefer not to say

Base: Eligible for bowel screening based on age/nation (50 and over in Eng, Scot and Wales; 60 and over in NI)
Question type: ***Single***
#Question display logic:
if (age_test > 59 and profile_GOR==12) or (age_test > 49 and profile_GOR in [1,2,3,4,5,6,7,8,9,10,11])

[Q41] Will you complete the bowel screening kit next time you are sent one?
Please select one answer.

<1>	Yes, definitely
<2>	Yes, probably
<3>	No, probably not
<4>	No, definitely not
<5>	I’m not eligible to receive a kit in the future
<6>	Don’t know
<7>	Prefer not to say

Base: Have been sent a kit/DK/refused
Question type: **Multiple**
#row order: randomize
#Question display logic:
If [Q40] - Yes or No or Don't know or Prefer not to say, is selected [if Q40 in [1,2,5,6]]

[Q42] Thinking about the last time you received a bowel cancer screening poo test kit, which, if any, of the following put you off completing it?
Please select all that apply.

<1>	I had other more important things to worry about than bowel screening	<9>	I was too afraid of having treatment if I was found to have cancer
<2>	I didn't have any symptoms of bowel cancer	<11>	I worried about putting extra strain on the NHS / health services
<3>	I was too busy to complete the poo test kit	<17>	After thinking about bowel cancer screening, I decided that the harms of taking part outweigh the benefits
<4>	I found it too difficult to complete the poo test kit	<12 fixed>	Other (open [Q42_12_open]) [open]
<5>	I don't think that I am at risk of developing bowel cancer	<13 fixed xor>	I have never received a bowel cancer screening poo test kit
<6>	I found it too messy to complete the poo test kit	<14 fixed xor>	Prefer not to say
<7>	I found it too embarrassing to complete the poo test kit	<15 fixed xor>	Nothing put me off completing it
<8>	I was too frightened of what the poo test might find	<16 fixed xor>	I don't remember

Question type: **Text**

Please note: Screening is for people who don't have symptoms. If you have symptoms, please speak to your GP.

Question type: **Text**

The next questions are about breast cancer screening. The breast screening is offered to women, some transgender men and some non-binary people aged between 50 and 70 in the UK.

YouGov

Base: Female/refused and eligible for breast screening based on age/nation
Question type: ***Single***
#Question display logic:
if (S1 in [2,3] and age_test > 49 and profile_GOR in [1,2,3,4,5,6,7,8,9]) or (S1 in [2,3] and age_test > 49 and (profile_GOR==11 or profile_GOR==10 or profile_GOR==12))

[Q43] Did you go for breast cancer screening the last time you were invited?
Please select one answer.

<1>	Yes
<2>	No
<3>	I have never been invited
<4>	I am not eligible
<5>	Don’t know
<6>	Prefer not to say

Base: Female/refused and eligible for breast screening based on age/nation
Question type: ***Single***
#Question display logic:
if (S1 in [2,3] and age_test > 49 and profile_GOR in [1,2,3,4,5,6,7,8,9]) or (S1 in [2,3] and age_test > 49 and (profile_GOR==11 or profile_GOR==10 or profile_GOR==12))

[Q44] Will you go for breast cancer screening next time you are invited?
Please select one answer.

<1>	Yes, definitely
<2>	Yes, probably
<3>	No, probably not
<4>	No, definitely not
<5>	I’m not eligible to be invited in the future
<6>	Don’t know
<7>	Prefer not to say

Base: Have been invited for screening/DK/refused
Question type: ***Multiple***
#row order: randomize #Columns: 2
#Question display logic:
If [Q43] - Yes or No or Don’t know or Prefer not to say, is selected [if Q43 in [1,2,5,6]]

[Q45] Thinking about the last time you were invited for breast screening, which, if any, of the following put you off going?
Please select all that apply.

<1>	I was worried that breast screening might be painful	<15>	I had to phone up and arrange the appointment myself
<2>	I didn’t have any symptoms of breast cancer	<19>	I have found breast cancer screening painful when I have been before

<3>	I had other more important things to worry about than breast screening	<22>	I didn't want a man to carry out the screening test
<4>	I don't think that I am at risk of breast cancer	<20>	I worried about having to take my clothes off or that too much skin would be showing
<5>	I was too embarrassed to go for breast screening	<23>	I could not afford to cover the costs related to having an appointment (e.g. transport, childcare, reduced pay/earnings)
<6>	I was too frightened of what the test might find	<24>	I worried my pay/earnings would be affected if I needed to have further tests or treatment
<7>	I was worried about catching COVID-19 if I went for screening	<16 fixed xor>	Nothing put me off going
<21>	After thinking about breast cancer screening, I decided that the harms of taking part outweigh the benefits	<17 fixed xor>	I don't remember
<9>	The appointment was too far away from my home	<18 fixed>	Other (open [Q45_18_open]) [open]
<10>	I had symptoms that might have been related to COVID-19	<11 fixed xor>	I have never been invited for breast screening
<13>	I found it difficult to get an appointment at a convenient time	<12 fixed xor>	Prefer not to say
<14>	I worried about putting extra strain on the NHS / health services		

Question type: ***Text***

Thank you for taking part in this survey. If you would like more information about cancer, you can visit:

Cancer Research UK's website here or call the freephone Cancer Research UK nurse helpline on 0808 800 4040 if you would like to speak to a cancer nurse.

Macmillan's website: here

The NHS website: here
